# Barriers and facilitators to the implementation of orthodontic mini implants in clinical practice: a systematic review

**DOI:** 10.1186/s13643-016-0336-z

**Published:** 2016-09-23

**Authors:** Reint Meursinge Reynders, Laura Ronchi, Luisa Ladu, Nicola Di Girolamo, Jan de Lange, Nia Roberts, Sharon Mickan

**Affiliations:** 1 Department of Oral and Maxillofacial Surgery, Academic Medical Center, University of Amsterdam, Meibergdreef 9, 1105 AZ Amsterdam, The Netherlands; 2 Via Matteo Bandello 15, 20123 Milan, Italy; 3 Department of Veterinary Sciences, University of Bologna, Via Tolara di Sopra 50, 40064 Ozzano dell’Emilia, BO Italy; 4 Department of Oral and Maxillofacial Surgery, Academic Medical Center and Academisch Centrum Tandheelkunde Amsterdam (ACTA), University of Amsterdam, Meibergdreef 9, 1105 AZ Amsterdam, The Netherlands; 5 Bodleian Health Care libraries, Cairns Library Level 3, John Radcliffe Hospital, University of Oxford, Oxford, OX3 9DU UK; 6 Department of Allied Health, Gold Coast Health and Griffith University, Queensland, QLD 4222 Australia

**Keywords:** Mini implant, Screw, Orthodontics, Implementation, Knowledge translation, Barriers, Facilitators, Contacting authors, Systematic review

## Abstract

**Background:**

Numerous surveys have shown that orthodontic mini implants (OMIs) are underused in clinical practice. To investigate this implementation issue, we conducted a systematic review to (1) identify barriers and facilitators to the implementation of OMIs for all potential stakeholders and (2) quantify these implementation constructs, i.e., record their prevalence. We also recorded the prevalence of clinicians in the eligible studies that do not use OMIs.

**Methods:**

Methods were based on our published protocol. Broad-spectrum eligibility criteria were defined. A barrier was defined as any variable that impedes or obstructs the use of OMIs and a facilitator as any variable that eases and promotes their use. Over 30 databases including gray literature were searched until 15 January 2016. The Joanna Briggs Institute tool for studies reporting prevalence and incidence data was used to critically appraise the included studies. Outcomes were qualitatively synthesized, and meta-analyses were only conducted when pre-set criteria were fulfilled. Three reviewers conducted all research procedures independently. We also contacted authors of eligible studies to obtain additional information.

**Results:**

Three surveys fulfilled the eligibility criteria. Seventeen implementation constructs were identified in these studies and were extracted from a total of 165 patients and 1391 clinicians. Eight of the 17 constructs were scored by more than 50 % of the pertinent stakeholders. Three of these constructs overlapped between studies. Contacting of authors clarified various uncertainties but was not always successful. Limitations of the eligible studies included (1) the small number of studies; (2) not defining the research questions, i.e., the primary outcomes; (3) the research design (surveys) of the studies and the exclusive use of closed-ended questions; (4) not consulting standards for identifying implementation constructs; (5) the lack of pilot testing; (6) high heterogeneity; (7) the risk of reporting bias; and (8) additional shortcomings. Meta-analyses were not possible because of these limitations. Two eligible studies found that respectively 56.3 % (952/1691) and 40.16 % (439/1093) of clinicians do not use OMIs.

**Conclusions:**

Notwithstanding the limitations of the eligible studies, their findings were important because (1) 17 implementation constructs were identified of which 8 were scored by more than 50 % of the stakeholders; (2) the various shortcomings showed how to improve on future implementation studies; and (3) the underuse of OMIs in the selected studies and in the literature demonstrated the need to identify, quantify, and address implementation constructs. Prioritizing of future research questions on OMIs with all pertinent stakeholders is an important first step and could redirect research studies on OMIs towards implementation issues. Patients, clinicians, researchers, policymakers, insurance companies, implant companies, and research sponsors will all be beneficiaries.

**Electronic supplementary material:**

The online version of this article (doi:10.1186/s13643-016-0336-z) contains supplementary material, which is available to authorized users.

## Background

Getting effective healthcare innovations into practice is often suboptimal [1–3]. This implementation issue also applies to orthodontic mini implants (OMIs) because surveys worldwide have shown that many clinicians rarely or never use these devices [4–8] notwithstanding their promising success rates, effectiveness, and applicability [9–11]. To understand the causes of this problem, it is important to identify the barriers and facilitators to the implementation of OMIs in clinical practice. This systematic review identified and quantified these implementation constructs.

Most orthodontic treatment plans need some form of anchorage to counteract the reciprocal forces of orthodontic tooth movement [12]. Numerous anchorage systems have been developed for this purpose. They generally apply forces to groups of teeth or use extra-oral traction to the neck or cranium. These techniques are effective, but they can still cause a loss of anchorage, have a limited area of application, and often depend on the constant collaboration of the patient [12]. OMIs are not limited by most of these characteristics; they can be implemented in a wide variety of orthodontic treatment plans and can be used in the maxilla and mandible for long periods of time [10]. The most frequently used OMIs are machine surfaced bone screws with a diameter of 1.3–2 mm and a length of 6–10 mm [11]. Both single and multiple OMIs with or without connecting plates are used for anchorage purposes. After insertion, OMIs are usually loaded immediately with orthodontic forces, and they are removed after the completion of the orthodontic treatment objectives.

Since the introduction of OMIs in 1997 by Kanomi [13], the number of publications on these devices has increased exponentially [14] and systematic reviews on OMIs have recorded promising low implant failure rates [11, 15] and favorable effectiveness [9, 10]. Numerous orthodontic companies have been founded and presentations of orthodontic treatments with OMIs have become the norm at orthodontic meetings [16]. International orthodontic conferences are even organized that focus exclusively on treatment with OMIs [17].

However, surveys in the USA, India, Germany, and England showed that many orthodontists never or rarely use these devices [4–8, 18–20]. This knowledge-to-action (KTA) gap, which is the gap between evidence-based knowledge and the use of this information in practice, was unexpected for a technique that has been introduced almost 20 years ago and has received such high acclaims [3, 9–11, 13]. This KTA gap was also unexpected in the context that most orthodontic treatment plans need some form of anchorage [12]. In addition, more than 75 % of 108 surveyed doctors included OMIs in their treatment plan for a common orthodontic patient [21].

KTA gaps are not just limited to interventional procedures with OMIs but are common for a variety of medical conditions and are a global problem [22, 23]. Only a small fraction of healthcare innovations gets incorporated into practice, and it has been estimated that 45 % of patients in the USA do not receive recommended care [23, 24]. In recent years, interest in the causes of KTA gaps and strategies for dealing with them has increased dramatically [25]. Identifying and quantifying implementation constructs of healthcare interventions is an important step to address these issues.

We therefore developed a systematic review that identified and quantified barriers and facilitators to the implementation of OMIs in clinical practice. We applied this primary research objective to both demand-side stakeholders, i.e., orthodontic patients and their family members, and potential supply-side stakeholders, e.g., clinicians, office staff, clinic owners, researchers, guideline developers, policy makers, and implant companies. Barriers to the implementation of OMIs could refer to the invasiveness of the interventional procedure, learning a new technique, the adverse effects of interventions and the fear of complications, financial barriers, the large volume of research evidence, the lack of trust in research evidence, the applicability of the new health technology to a local context, etc. [5, 8, 26–28]. Facilitators to implementation of OMIs could refer to shorter treatment time, better outcomes, improved esthetics during orthodontic treatment, recommendations by patients who had undergone treatment with OMIs, etc. [5, 29, 30]. Research data on the identified and quantified implementation constructs can be subsequently consulted to develop tailored strategies to deal with them.

For our secondary objectives, we recorded the prevalence of clinicians that do not use OMIs in clinical practice. This statistic was extracted from the studies that were identified as “eligible” for our primary research question. During our scoping searches, we identified no systematic reviews that addressed our primary and secondary research questions on the implementation of OMIs.

### Objectives

The following objectives of this systematic review were defined:

#### Primary objective

- To identify and quantify barriers and facilitators to the implementation of OMIs for all potential stakeholders such as patients and their family members, clinicians, office staff, clinic owners, researchers, guideline developers, policy makers, and implant companies.

#### Secondary objective

- To record the prevalence of clinicians that do not use OMIs in the studies that were selected for the primary objectives. This statistic represents the knowledge-to-action gap for these stakeholders.

## Methods

We applied the methods that were described previously in our published protocol [14]. Various sources were consulted to develop these methods: (1) established conceptual models for assessing barriers and facilitators to the implementation of knowledge use [22, 25, 26, 31, 32]; (2) guidelines and handbooks for designing and reporting qualitative and quantitative systematic reviews [33–38]; (3) guidelines and checklists for reporting research studies [39]; (4) systematic reviews that were specifically designed to identify implementation constructs and addressed similar research objectives as our research study [27, 28, 40–44]; and (5) systematic reviews and their protocols on OMIs that we have published previously [14, 45–48].

The characteristics and application of these sources are further explained in the pertinent sections of this systematic review. Differences between our protocol and the final systematic review are reported in the “Results” section together with the rationale and the consequences of these modifications. We adopted the PRISMA 2009 Statement for reporting systematic reviews and presented this manuscript according to the order of this guideline (Additional file 1)[36, 49]. This systematic review was not registered in the PROSPERO database because our research questions are not covered by the inclusion criteria of this register [50].

### Eligibility criteria

We applied the eligibility criteria that were outlined in our published protocol [14]. These criteria are summarized under here.

#### Study designs

For the primary objectives, we defined the following eligibility criteria:

- Studies that collected original data on identified barriers and facilitators to the implementation of OMIs in clinical practice were eligible. To avoid exclusion of pertinent studies, we did not set eligibility criteria for specific research designs. We expected to find interviews, focus groups, surveys, and questionnaires as the most common eligible research designs.
- Studies that addressed our primary research question as their primary or secondary objectives were eligible. For example, studies that identified barriers and facilitators to the implementation of OMIs in clinical practice as a part of a larger mixed methods model were eligible.

For the secondary objectives, we defined the following eligibility criteria:

- Only quantitative studies, for example, surveys, that addressed the primary objectives of this systematic review were eligible.

#### Stakeholders (participants)

We applied broad-spectrum selection criteria that included all potential demand- and supply-side stakeholders. The former category refers to patients undergoing the interventional procedure and the pertinent family members. The latter category refers to the following stakeholders: clinicians, clinic owners, researchers, office staff, guideline developers, policy makers, and implant companies.

#### Interventions

Interventions that used single or multiple implants with diameters smaller than 2.5 mm for orthodontic anchorage objectives were eligible. We applied no restrictions to the length or design of the implant, its connection to plates, its location of insertion, the type of method for implant insertion, the type of orthodontic loading, and the type of implant maintenance after its insertion. Interventions with OMIs on patients of any age, sex, or demographics were eligible.

#### Outcomes

Any type of barrier or facilitator to the use of OMIs in clinical practice was selected as our primary outcome. A barrier was defined as any variable that impedes or obstructs their use. A facilitator was defined as any variable that eases and promotes the use of OMIs. Barriers and facilitators were eligible when they were described as implementation constructs by the eligible stakeholders [44]. For example, patient’s perceptions of the interventional procedure or assessments of health experiences such as pain and discomfort during implant insertion were not considered as eligible outcomes when they were not specifically defined as barriers to the use of OMIs by these patients. These eligibility criteria avoid mislabelling of implementation constructs during qualitative analyses as a result of bias or misinterpretation of outcomes by systematic reviewers.

#### Setting and language

No setting and language restrictions were applied.

### Information sources and search

Information sources were searched from 1 January 1997, the year of the first publication on OMIs, until 15 January 2016 [13].

- The following general and subject-specific electronic databases were searched: Google Scholar Beta, PubMed (MEDLINE), EMBASE (Ovid), Cochrane Central Register of Controlled Trials (CENTRAL), CINAHL, PsycINFO, Sociological Abstracts, and PROSPERO [51–55].
- The “Related Articles” feature in PubMed was consulted.
- The following Web of Science Core collection citation indexes were searched: Science Citation Index Expanded (SCI-EXPANDED); ARTS and Humanities Citation Index (A&HCI); and Social Sciences Citation Index (SSCI) [53, 56, 57].
- A series of national and regional databases was also searched: African Index Medicus, African Journals online (AJOL), Informit Health Collection, Index Medicus for the Eastern Mediterranean Region, IndMED, KoreaMed, LILACS, Index Medicus for the South-East Asia Region (IMSEAR), Western Pacific Region Index Medicus (WPRIM) [53, 56].
- We also consulted “other resources” which included the gray literature, reference lists, and hand-searches of key journals. A detailed list of these resources was presented in our published protocol [14].
- We also contacted pertinent stakeholders on our topic of interest.

Methods to find pertinent subject headings and key words were adopted from our previous systematic reviews and protocols on OMIs [14, 45–48]. An information specialist (NR) in computerized searches of healthcare publications assisted with the development of the search strategies. In our protocol, we presented the full electronic search strategies for both PubMed and Google Scholar Beta [14]. For PubMed, we used the following search strategy: (orthodontics OR orthodontic OR orthodontist OR orthodontists) (implant OR implants OR “mini implant” OR “mini implants” OR screw OR screws OR “mini screw” OR “mini screws” OR “miniscrew” OR “miniscrews” OR “microscrew” OR “temporary anchorage device”).

### Study selection

Eligible studies for this systematic review were selected independently by three review authors (RMR, LR, and LL). Disagreements between these operators on the eligibility of an article were resolved through rereading of the pertinent article, discussions, and if necessary through contacting its authors [49]. In the case of persistent disagreements, a fourth author (NDG) was asked to arbiter.

The selection of studies was summarized in a PRISMA flow diagram [36, 49]. Excluded articles were listed in a table with the reasons for their exclusion. A detailed description of the procedures for selecting studies, contacting authors, and for assessing multiple publications of the same research data and dealing with these issues were presented in our published protocol [14]. All study selection procedures were conducted according to this protocol.

### Data collection process and data items

Prior to the formal study selection and data extraction process, a list of “potential” barriers and facilitators to the implementation of OMIs was developed by the three reviewers (RMR, LR, and LL). This list was developed through the assessment and discussions of three groups of publications: (1) systematic reviews that focused on the identification of barriers and facilitators to the implementation of health-related issues and technologies [27, 28, 40–44]; (2) conceptual models for assessing barriers and facilitators to knowledge use [25, 26, 31, 32, 58]; and (3) our previous systematic reviews and protocols on OMIs [14, 45–48]. This list of “potential” barriers and facilitators was created prior to the study selection and data extraction process and was used to calibrate the three reviewers and to increase their background knowledge on implementation constructs. Our list of “potential” implementation constructs was not used as a reference checklist during the study selection and data collection process because this could have resulted in the inappropriate exclusion of “unexpected” barriers and facilitators to the implementation of OMIs in clinical practice.

For the development of the data collection forms, we explored (1) the reporting checklists of pertinent research designs of the Equator network [39]; (2) the data extraction forms of previous systematic reviews and protocols on OMIs [14, 45–48]; and (3) the three groups of publications that were used to develop the list of potential implementation constructs [25–28, 31, 32, 40–48, 58]. Pertinent items for the extraction of data for the secondary research question were also assessed during this research phase. Extracted data items for our primary and secondary objectives include: the source, eligibility, duplicate publication, the study design, selection procedures, stakeholders, the setting, interventions, outcomes, flow and timing, adverse effects, withdrawals and missing outcomes, the funding, and miscellaneous data of the selected studies [36, 38]. Many of these items were further subdivided and all extracted entries were listed in tables. Descriptions of each item were presented in these tables and entries that could bias the outcomes were also recorded.

All data extraction procedures were conducted independently by the three aforementioned operators, who are experienced systematic reviewers and topic experts. Disagreements on extracted items were resolved through rereading and discussions and if necessary an arbitrator (NDG) was consulted to adjudicate these disagreements. Our pilot-tested data extraction forms and a detailed description of our data collection procedures were given in the published protocol of this systematic review [14].

### Outcomes and prioritization

#### Primary outcomes

- The primary outcomes were all barriers and facilitators to the implementation of OMIs in clinical practice identified by all demand-and supply-side stakeholders. When given, we recorded also the prevalence of implementation constructs among subgroups of pertinent stakeholders.
- Pertinent stakeholders were defined in Table 1 and were divided in “users” and “non-users.” Potential subgroups for which outcomes were recorded are presented in Table 1.

**Table 1:** Potential subgroups for which outcomes were recorded

General subgroups	Specific subgroups
Research design	Surveys: outcomes obtained through surveys (questionnaires) of stakeholders Interviews: outcomes obtained from interviews with stakeholders Focus groups: outcomes obtained from focus groups with stakeholders
Stakeholders	*Demand-side stakeholders:* orthodontic patients and their family members *Supply-side stakeholders:* clinicians, office staff, clinic owners, researchers, guideline developers, policy makers, implant companies, etc. *Users/non-users*: both demand- and supply-side stakeholders can be further subdivided in those that have used OMIs previously (users) and those that have never used these devices (non-users)
Interventions	*Specified interventions:* these interventions refer to a specific phase or type of the interventional procedure. Phases of the intervention refer to the anesthetics, implant insertion, orthodontic treatment with OMIs, implant removal, or the healing phase. Types of interventions refer to the implant type and dimensions, number of implants, use of plates, the surgical procedure, implant location, timing and forces of orthodontic loading, etc. [48]. “*Non specified*” *interventions:* these interventions refer to “any orthodontic treatment with OMIs.” Additional information on the specific phase or type of the interventional procedure is not provided
Time points	*Pre-intervention recordings*, i.e., recordings of outcomes prior to the interventional procedure *Immediate post-intervention recordings*, i.e., recordings of outcomes within 2 weeks after the completion of the interventional procedure *Long-term post-intervention recordings*, i.e., recordings of outcomes more than 2 weeks after the completion of the interventional procedure
Setting/country	*Private practice:* stakeholders treated or working in a private practice *University setting:* stakeholders treated or working in a university clinic Country: stakeholders treated or working in a specific country
- A barrier is defined as any variable that impedes or obstructs the use of OMIs. A facilitator is defined as any variable that eases and promotes their use.
- The pre-intervention recording of barriers or facilitators to a “specified” intervention with OMIs for “non-user” clinicians or for patients that have not undergone this intervention previously in any type of setting or research design was our “preferred” primary outcome.
- The prevalence of identified barriers and facilitators among the surveyed or interviewed pertinent stakeholders was calculated as follows:
  $$ \begin{array}{l}\mathrm{Prevalence}\ \mathrm{o}\mathrm{f}\ \mathrm{a}\mathrm{n}\ \mathrm{identified}\ \mathrm{barrier}\ \mathrm{o}\mathrm{r}\ \mathrm{f}\mathrm{a}\mathrm{cilitator} = \mathrm{t}\mathrm{he}\ \mathrm{n}\mathrm{umber}\ \mathrm{o}\mathrm{f}\ \mathrm{s}\mathrm{t}\mathrm{a}\mathrm{keholders}\ \mathrm{t}\mathrm{hat}\ \mathrm{s}\mathrm{cored}\\ {}\mathrm{a}\ \mathrm{particular}\ \mathrm{construct}\ \mathrm{a}\mathrm{s}\ \mathrm{a}\ \mathrm{barrier}\ \mathrm{o}\mathrm{r}\ \mathrm{f}\mathrm{a}\mathrm{cilitator}\ \mathrm{t}\mathrm{o}\ \mathrm{t}\mathrm{he}\ \mathrm{implementation}\ \mathrm{o}\mathrm{f}\ \mathrm{OMIs}\ \mathrm{in}\ \mathrm{clinical}\\ {}\mathrm{practice}/\mathrm{the}\ \mathrm{t}\mathrm{o}\mathrm{t}\mathrm{a}\mathrm{l}\ \mathrm{n}\mathrm{umber}\ \mathrm{o}\mathrm{f}\ \mathrm{s}\mathrm{t}\mathrm{a}\mathrm{keholders}\ \mathrm{t}\mathrm{hat}\ \mathrm{s}\mathrm{cored}\ \mathrm{o}\mathrm{n}\ \mathrm{t}\mathrm{he}\ \mathrm{r}\mathrm{o}\mathrm{l}\mathrm{e}\ \mathrm{o}\mathrm{f}\ \mathrm{t}\mathrm{his}\ \mathrm{particular}\ \mathrm{construct}\\ {}\mathrm{a}\mathrm{s}\ \mathrm{a}\ \mathrm{barrier}\ \mathrm{o}\mathrm{r}\ \mathrm{f}\mathrm{a}\mathrm{cilitator}\ \mathrm{t}\mathrm{o}\ \mathrm{t}\mathrm{he}\ \mathrm{implementation}\ \mathrm{o}\mathrm{f}\ \mathrm{OMIs}\ \mathrm{in}\ \mathrm{clinical}\ \mathrm{practice}.\end{array} $$
  This prevalence was presented for example as 30/50.
- In our published protocol, we presented additional information on (1) defining primary outcomes; (2) the procedures to extract and categorize primary outcomes; and (3) anticipated exemplary tables of categorized implementation constructs [14, 38].

#### Secondary outcomes

- The secondary outcome was the prevalence of clinicians that do not use OMIs and represents the knowledge-to-action gap. This statistic was calculated as follows:
  $$ \begin{array}{l}\mathrm{The}\ \mathrm{prevalence}\ \mathrm{of}\ \mathrm{clinicians}\ \mathrm{that}\ \mathrm{do}\ \mathrm{not}\ \mathrm{use}\ \mathrm{OMIs} = \mathrm{the}\ \mathrm{number}\ \mathrm{of}\ \mathrm{clinicians}\ \mathrm{that}\ \mathrm{do}\ \mathrm{not}\ \mathrm{use}\\ {}\mathrm{OMIs}/\mathrm{The}\ \mathrm{total}\ \mathrm{number}\ \mathrm{of}\ \mathrm{surveyed}\ \mathrm{clinicians}\ \mathrm{that}\ \mathrm{reported}\ \mathrm{on}\ \mathrm{the}\ \mathrm{use}\ \mathrm{of}\ \mathrm{OMIs}\ \mathrm{in}\ \mathrm{clinical}\ \mathrm{practice.}\end{array} $$

Information that could give further insights in the understanding of the knowledge-to-action gap, e.g., the number of implants placed per clinician per year, was also recorded.

### Risk of bias in individual studies

According to our protocol [14], we applied critical appraisal instruments that were specific for the type of research design used in the eligible studies. In this review, we adopted The Joanna Briggs Institute critical appraisal tool for quantitative studies that report prevalence and incidence data [35, 42, 59]. This instrument has been specifically developed for questionnaires and surveys. Differences between reviewers in scoring these tools were resolved through discussions. A fourth reviewer (NDG) was called upon in the case of disagreement between reviewers. Authors of eligible studies were contacted in the case of persistent disagreements on appraisal scores.

The critical appraisal scores for each selected study were listed in tables and for each appraisal tool separately [34, 35]. We calculated the prevalence of “yes” scores (number of “yes”/number of articles) for each individual appraisal question [42]. No attempts were made to calculate overall appraisal scores. The potential influence of each of the scored answers on the outcomes of each selected study was weighted during the data synthesis and was used to assess the overall strength of evidence of the review (see “Confidence in cumulative evidence”) [38]. Additional details on the procedures for the assessment of risk of bias and the instruments for assessing the methodological quality of studies were presented in our published protocol [14].

### Synthesis of results

#### Criteria for a quantitative synthesis

We only conducted meta-analyses for our primary and secondary outcomes when (1) the risk of bias in the eligible studies was low; (2) outcomes between studies were consistent; (3) publication bias was low; (4) a high number of studies was included; and (5) heterogeneity was low [60–62]. Forest plots were used to display the dispersion of the even rates of both primary and secondary outcomes. Comprehensive meta-analysis (CMA) software was used to conduct all statistical analyses in this systematic review [63, 64].

#### Unit-of-analysis issues and missing data

To deal with unit-of-analysis issues, we assessed whether all participants underwent the same intervention, multiple interventions, and whether outcomes were recorded at different or multiple time points [61]. Our strategies for dealing with missing data were presented in our published protocol [14].

#### Qualitative synthesis

According to the PRISMA-P 2015 statement, we undertook a systematic narrative (qualitative) synthesis even when criteria for conducting quantitative syntheses were fulfilled [38]. Our narrative synthesis was conducted systematically and transparently to reduce the potential for bias [65]. We refrained from vote counting, i.e., counting those studies that yielded a significant result and those that did not [66, 67]. As suggested by the PRISMA-P 2015 statement [38], we adopted the “established methods” for conducting systematic narrative syntheses according to the guidance of the Centre for Reviews and Dissemination (CRD) [65]. The CRD framework for conducting such a synthesis consists of four phases: (1) developing a theory why and how each barrier or facilitator could affect the implementation of OMIs for each linked stakeholder; (2) developing an initial synthesis of the findings of the eligible studies; (3) exploring relationships within and between studies; and (4) assessing the robustness of the synthesized evidence [65]. These steps do not have to be conducted exactly according to the order of this framework and were conducted iteratively by the three topic experts (RMR, LR, LL) [65]. Disagreements were resolved through discussions, and persistent disagreements were resolved through the arbitrage of a fourth author (NDG) or through contacting pertinent authors.

### Risk of bias across studies

#### Meta-biases and confidence in the cumulative evidence

Meta-bias refers to the biased selection of research data and covers both reporting bias (selective outcome reporting) and publication bias [38]. Methods to assess the presence and impact of both biases and strategies for dealing with them were described in detail in our published protocol [14, 54, 68–70].

For the assessment of the strength of the body of evidence, we consulted the guidelines described by the GRADE approach [71]. The robustness of the synthesized evidence depends on (1) the number and size of the eligible studies; (2) within and between study diversity; (3) risk of bias assessments (magnitude and direction); (4) the consistency of the outcomes between studies; (5) the magnitude of the outcomes; and (6) the presence of publication bias. To assess the robustness of identified evidence we (1) weighed the role of these variables; (2) revisited the data collection forms and the critical appraisal tools to assess whether items have been overlooked; and (3) contacted authors to obtain additional information. We did not score the “levels of evidence” according to the GRADE approach. Our research questions do not qualify for this approach because they do not address questions about interventions, management strategies, or policies [72].

### Additional analyses

#### Investigation of heterogeneity

We considered three sources of heterogeneity: methodological, clinical, and other sources of heterogeneity [61, 73]. These sources were selected a priori based on information from previous systematic reviews on this research topic and through discussions between the reviewers [45–48, 74]. Our “a priori” defined potential sources of heterogeneity were listed in our published protocol [14]. The type of stakeholders, i.e., patients, clinicians, and office staff, was excluded as a source of diversity because outcomes were analyzed separately for each type of stakeholder. We reported when “post hoc” defined sources of heterogeneity were investigated.

The presence of statistical heterogeneity was investigated by calculating Cochran’s Q, the degrees of freedom based on the number of eligible studies, and the pertinent *p* value [64, 75–77]. We also calculated the following statistics: Kendall’s *τ* ^2^, *τ*, and *I* ^2^ [64, 75, 77–80]. These calculations, their use, and strategies for dealing with heterogeneity were explained in our published protocol [14, 61].

#### Subgroup analysis, meta-regression, and sensitivity analysis

Our protocol described our planned methods for conducting subgroup analyses, meta-regressions, and sensitivity analyses [14]. Criteria, rationales, and caveats for undertaking such research procedures were also outlined in this protocol [61, 81, 82].

### Contacting authors

Authors of pertinent primary research studies were contacted to obtain additional information on (1) the eligibility of specific research studies and (2) unclear or missing data in primary research studies. The methods for this research procedure were described in detail in our published protocol [14].

### Differences between the protocol and the review

We reported all changes in the methods during the conduct of this research study compared with those planned in the protocol. We described the type, timing, and the rationale of each of these modifications. We also reported the consequences of these changes on the direction, the magnitude, and the validity of the outcomes [83].

## Results

### Study selection

**Fig. 1:**
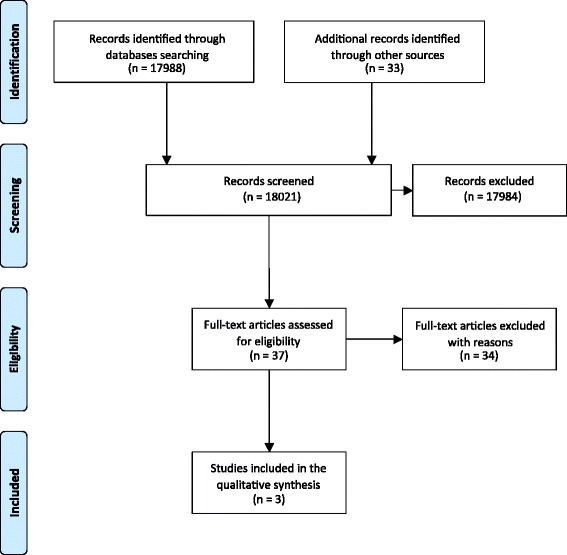
PRISMA flow diagram of the study selection procedures [84]

The outcomes of the searches of the various information sources were summarized in a PRISMA flow diagram (Fig. 1) [84]. A total of 18,021 records with overlap were identified during the searching procedures. The retrieved records for each data source together with the search dates were listed in Additional file 2. We identified 37 articles, whose full texts were assessed for eligibility. Three of these studies fulfilled the selection criteria. The 34 excluded articles with their references were listed together with the rationale for their exclusion in Additional file 3. Most of these studies were excluded because patient health experiences or data on the use of OMIs were recorded but not implementation constructs. The selection procedures of eligible studies were conducted in complete agreement between all three reviewers.

### Study characteristics

The characteristics of the three included studies were summarized in Tables 2, 3, 4, and 5. Information obtained through contacting authors was listed in red-type face in these tables. All eligible studies were surveys that used questionnaires as their research tools (Table 2). All survey questions in the selected studies were closed-ended, undefined, and not validated or pilot tested (Table 2). Clinicians and patients were the only types of stakeholders that were surveyed in these studies (Table 3). The sample sizes and the response rates of the included studies varied widely. In the study by Zawawi [85], all (165/165) surveyed stakeholders responded to questions on barriers and facilitators (Table 3). In the studies of Meeran et al. [8] and Bock and Ruf [5], the overall response rates of the questionnaires were respectively 80.5 % (1691/2100) and 47.9 % (1177/2459). Subpopulations of non-users of OMIs in these latter studies were subsequently surveyed on implementation constructs (Table 3). In the study by Bock and Ruf [5], 84 of the 1177 questionnaires were excluded because of missing or flawed information.

**Table 2:**
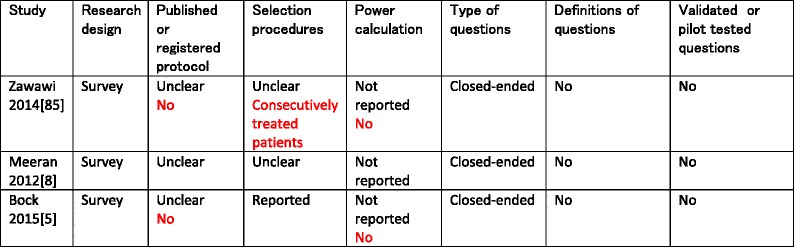
Characteristics of research methods

Items in black-type face represent those of the published manuscript. Items in red-type face represent those obtained through contacting the authors of the pertinent manuscript

**Table 3:**
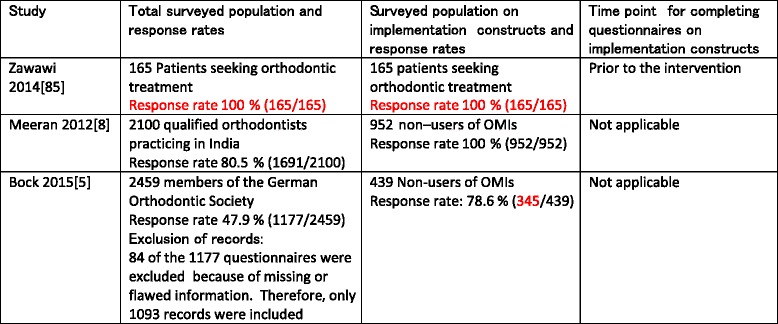
Response rates of surveyed populations and time points for completing questionnaires on implementation constructs

Items in black-type face represent those of the published manuscript. Items in red-type face represent those obtained through contacting the authors of the pertinent manuscript

**Table 4:**
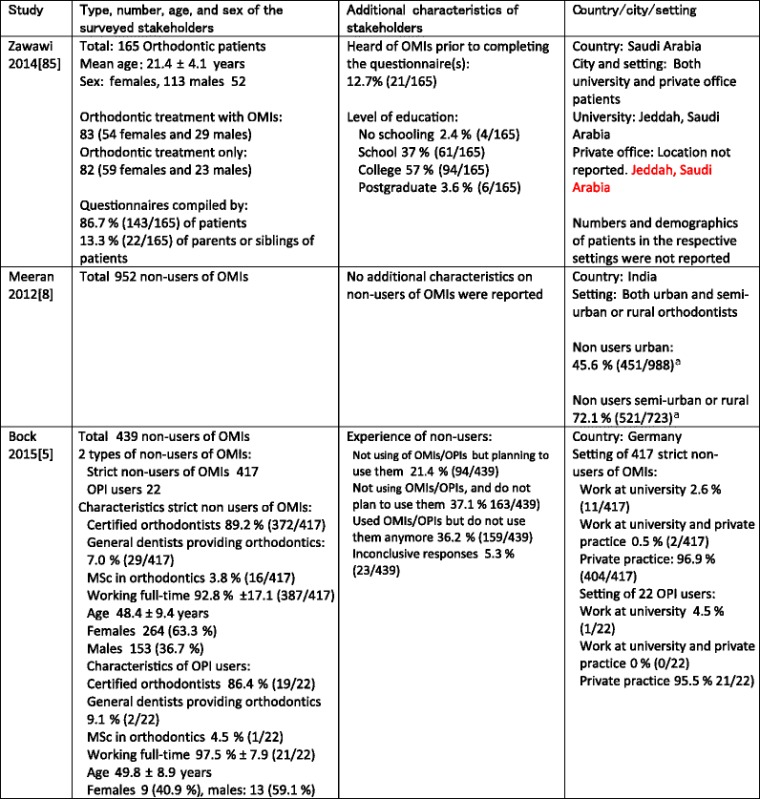
Type of stakeholders surveyed on implementation constructs and their settings ^a^ ^b^

Items in black-type face represent those of the published manuscript. Items in red-type face represent those obtained through contacting the authors of the pertinent manuscript. For both the studies by Zawawi et al. (2014) [85] and Meeran et al. (2012) [8], these data are also representative for their responding populations because response rates were 100 %. For the study by Bock et al. (2015) [5], these data were not representative for the responding population because response rates were 78.6 % (345/439)

*OPI* osseointegrated palatal implant

^a^Numbers of non-users do not add up, i.e., 451 + 521 = 972 and not 952

**Table 5:** Type of interventions

Study	Definition of interventions	“Specified” or “Non specified” intervention	Plates	Type and number of OMIs	Location	Duration (weeks)
Zawawi 2014 [85]	Reported	Non specified intervention	Not reported	OrthoEasy^a^ Length 6 and 8 mm Diameter 1.8 mm	Maxilla and mandible	Not reported
Meeran 2012 [8]	Reported	Non specified intervention	Not reported	Not reported	Not reported	Not reported
Bock 2015 [5]	Reported	Non specified intervention	Not reported	Not reported	Not reported	Not reported

^a^OrthoEasy system (Forestadent, Pforzheim, Germany)

A total of 1556 stakeholders were surveyed on implementation constructs consisting of 165 patients [85] and 1391 clinicians, i.e., 952 in Meeran’s [8] and 439 in Bock’s and Ruf’s study [5] (Table 3). Response rates on implementation constructs were 100 % in the first two studies [8, 85] and 78.6 % (345/439) in the latter article [5]. Clarification on this latter prevalence was obtained through contacting Dr. Bock [5]. The 165 patients in Zawawi’s study [85] came from a university and a private practice setting in Saudi Arabia and were mostly females (113 females versus 52 males). Of these patients, 50.3 % (83/165) needed orthodontic treatment with OMIs and 12.7 % (21/165) had heard of OMIs prior to completing the questionnaires.

Questionnaires in Zawawi’s study [85] were compiled by 86.7 % (143/165) of the patients and by 13.3 % (22/165) of the parents or siblings of patients (Table 4). In the survey of Meeran et al. [8], 45.6 % (451/988) of clinicians in an urban setting and 72.1 % (521/723) of clinicians in a semi-urban or rural setting were the surveyed population of non-users of OMIs (Table 4). Because the total of clinicians in the different settings accounted for 1711 (988 + 723) stakeholders, which differed from the total of 1691 respondents, we asked the authors for clarification, but they did not respond.

The members of the German Orthodontic Society that were surveyed on implementation constructs in Bock and Ruf’s study [5] consisted of 439 non-users of OMIs, which were divided in 417 strict non-users (non-users of OMIs or osseointegrated palatal implants) and 22 users of osseointegrated palatal implants [5]. Additional characteristics on these stakeholders were listed in Table 4. These characteristics were representative for the total group (439) of non-users of OMIs and not for the 345 respondents of the non-users of OMIs. The type of interventions was presented in Table 5. All included studies defined the interventions with OMIs and referred to “non specified interventions” indicating “any type of orthodontic treatment with OMIs.”

### Risk of bias within studies

We used the Joanna Briggs Institute critical appraisal tool for studies that reported prevalence and incidence data because all eligible studies were surveys [35, 59]. No major discussions between the three reviewers were necessary to reach agreement on the appraisal scores. These outcomes were listed in Table 6. The prevalence of “yes” scores (number of “yes”/number of articles) for each individual appraisal question was listed in this table [42]. All eligible studies scored the same two appraisal questions as “no.” None of these three studies used a reference standard for assessing implementation constructs and serious confounding issues were identified in all studies. The rationales for the appraisal scores were described in Additional file 4, and all limitations of the included studies were summarized in Table 7. The influence of the critical appraisal scores on the overall strength of the evidence of this systematic review was discussed in the section “Synthesis of results”.

**Table 6:**
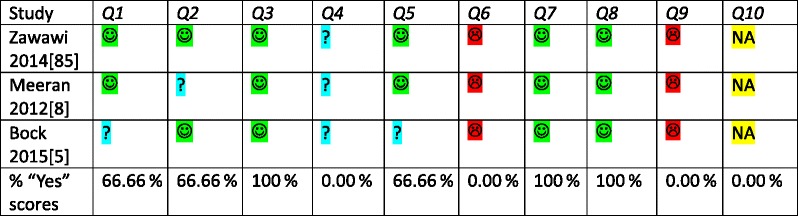
Tabular presentation of the scores of the Joanna Briggs Institute critical appraisal tool of prevalence and incidence data [35, 59]^a^

Critical appraisal criteria for quantitative studies [35]: (1) Was the sample representative of the target population? (2) Were study participants recruited in an appropriate way? (3) Was the sample size adequate? (4) Were the study subjects and the setting described in detail? (5) Was the data analysis conducted with sufficient coverage of the identified sample? (6) Were objective, standard criteria used for the measurement of the condition? (7) Was the condition measured reliably? (8) Was there appropriate statistical analysis? (9) Are all important confounding factors/subgroups/differences identified and accounted for? (10) Were subpopulations identified using objective criteria? Critical appraisal scores:  yes  no  unclear  not applicable

**Table 7:** Limitations of the included studies

Study	Limitations of the included studies
Zawawi 2014 [85]	• No published or registered protocol of the research study (risk of selective reporting) • The research design (survey) • Questionnaires with only closed-ended questions • Mostly non-defined questions • No pilot testing of research procedures • No reference was made to a reference standard for assessing implementation constructs. Survey instruments were not validated • Unclear selection procedures but were clarified through contacting the author • Unclear whether the answers of parents and siblings (13.3 % of the respondents) were representative for those of the stakeholders • The surveyed population was recruited from 2 different settings • Prior knowledge on OMIs of 12.7 % (21/165) of respondents • Additional unclear issues in Tables 2, 3, 4, and 5 and among the critical appraisal scores (Additional file 4)
Meeran 2012 [8]	• No published or registered protocol of the research study (risk of selective reporting) • The research design (survey) • Questionnaires with only closed-ended questions • Mostly non-defined questions • No pilot testing of research procedures • No reference was made to a reference standard for assessing implementation constructs. Survey instruments were not validated • Unclear selection procedures • Summing the total numbers of respondents in the different settings does not add up to the total number of respondents referred to in other parts of the results • The surveyed population was recruited from 2 different settings and were not equally distributed. Urban respondents were overrepresented in the total sample and had less non-users of OMIs compared with the non-urban population. This issue could have skewed outcomes • Additional unclear issues in Tables 2, 3, 4, and 5 and among the critical appraisal scores (Additional file 4)
Bock 2015 [5]	• No published or registered protocol of the research study was identified (risk of selective reporting) • The research design (survey) • Questionnaires with only closed-ended questions • Mostly non-defined questions • No pilot testing of research procedures • No reference was made to a reference standard for assessing implementation constructs. Survey instruments were not validated • Unclear terminology in the questionnaire had created confusion among respondents on the correct interpretation of questions. This misunderstanding could have resulted in an overestimation of all around users and OPI-only users and therefore an underestimation of MSC-only users, but this issue did not affect our populations of interest • The numerators and denominators were not completely clear in the published article and were confirmed through contacting the authors of this research study • Both strict non-users of OMIs and users of osseointegrated palatal implants were included among the respondents, i.e., 417 (95 %) strict non-users of OMIs and 22 (5 %) osseointegrated palatal implants users. Only strict non-users of OMIs would have been our preferred target population • Various types of experience among subgroups of non-users of OMIs • The 439 non-users were adequately described, but the characteristics of the 345 respondents on implementation constructs of these 439 non-users were not described • The response rate of the non-users of OMIs was 78.6 % (345/439). The rationale and the consequences of this dropout were not described • Additional unclear issues in Tables 2, 3, 4, and 5 and among the critical appraisal scores (Additional file 4)

### Outcomes of individual studies

The three included studies identified a total of 17 implementation constructs, 13 for clinicians and 4 for patients (Table 8). Fourteen of these constructs were barriers and 3 were facilitators. The prevalence of each barrier and facilitator to the use of OMIs was also listed in Table 8. The reporting of the numerators and denominators for these statistics in the study by Bock and Ruf [5] was unclear. We were able to confirm these numbers through contacting Dr. Bock.

**Table 8:**
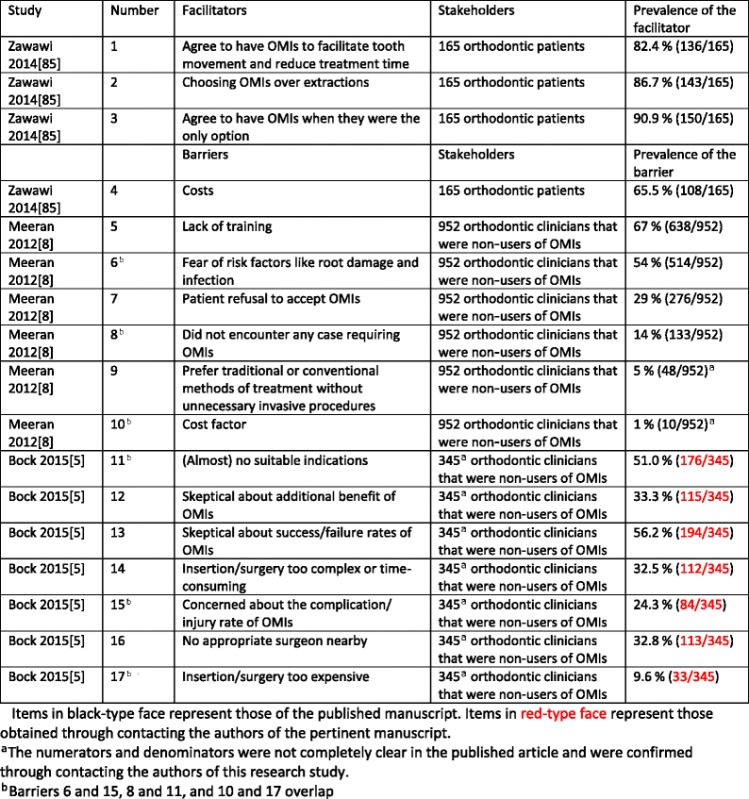
Barriers and facilitators to the implementation of OMIs^a^

Items in black-type face represent those of the published manuscript. Items in red-type face represent those obtained through contacting the authors of the pertinent manuscript

^a^The numerators and denominators were not completely clear in the published article and were confirmed through contacting the authors of this research study

^b^Barriers 6 and 15, 8 and 11, and 10 and 17 overlap

Only one included study surveyed patients on potential implementation constructs [85]. This study identified three facilitators (prevalence of 82.4 % (136/165), 86.7 % (143/165), 90.9 % (150/165)) and 1 barrier (prevalence 65.5 % (108/165)), to the use of OMIs (Table 8). The remaining two studies identified a total of 13 barriers to the use of OMIs for clinicians [5, 8] (Table 8). Three of these barriers overlapped between both studies. This overlap could not be assessed with 100 % certainty because none of the included studies defined their questions on implementation constructs. Lack of training (67 % (638/952) and fear of risk factors (54 % (514/952) were the two highest prevalence statistics for barriers in Meeran’s study [8]. (Almost) no suitable indications for OMIs 51 % (176/345) and skeptical about success/failure rates of OMIs 56.2 % (194/345) were the highest prevalence statistics for barriers in the study by Bock and Ruf [5]. The remaining nine implementation constructs were all scored by 1/3 or less of the respondents.

Two [5, 8] of the included studies addressed our secondary research question on the use of OMIs (Table 9). A total of 2784 (1691 + 1093) clinicians were surveyed on this question. The prevalence of clinicians that do not use OMIs was respectively 56.3 % (952/1691) [8] and 40.16 % (439/1093) [5]. The prevalence of non-urban non-users of OMIs, 72.1 % (521/723), was much higher than the prevalence of urban non-users, 45.6 % (451/988), in the study by Meeran et al. [8], but imprecision was identified in reporting the numerators and denominators (Table 9).

**Table 9:** Use of OMIs by clinicians

Study	Overall response rate to questionnaires	Prevalence of clinicians that do not use OMIs	Subgroup of non-users of OMIs
Meeran 2012 [8]	80.5 % (1691/2100)	56.3 % (952/1691)	Urban non-users, 45.6 % (451/988)^a^ Non-urban non-users, 72.1 % (521/723)^a^
Bock 2015 [5]	47.9 % (1177/2459)	40.16 % (439/1093)^b^	

^a^The numerators and denominators of non-users do not add up in the study by Meeran et al. (2012 [8]). Numerators, 451 + 521 = 972 and not 952. Denominators, 988 + 723 = 1711 and not 1691

^b^Eighty-four of the 1177 questionnaires were excluded because of missing or flawed information. Therefore, only 1093 records were included

### Risk of bias across studies (meta-biases)

Protocols of the included studies could not be found in the literature, and we therefore contacted the authors on this issue. Dr Zawawi [85] and Dr Bock [5] responded that protocols were not registered or published. Risk of selective outcome reporting was therefore assigned to these studies. Dr. Meeran and his co-author, Dr Venkatesh, did not respond to our questions [8]. Our assessments of publication bias were conditioned by the small number of eligible studies for each identified implementation construct [68]. Funnel plots were also not indicated for this reason.

### Synthesis of results

#### Quantitative synthesis

Eleven of the 17 identified implementation constructs were only found in single studies and therefore did not qualify for a quantitative synthesis. The remaining 6 constructs overlapped between 2 studies [5, 8] and were barriers to the implementation of OMIs for clinicians. Although the prevalence statistics of these barriers (clinician’s concerns regarding risks of using OMIs, clinician’s concerns regarding indications of OMIs, clinician’s concerns regarding the costs of OMIs) could be summarized as pairs in meta-analyses, we decided not to conduct such syntheses because (1) definitions of implementation constructs were not presented, and it was therefore impossible to assess whether we were synthesizing the same outcomes; (2) the number of included studies was small (just two); (3) outcomes between studies were inconsistent; (4) clinical and methodological heterogeneity of the included studies was high or unclear for numerous variables [60–62]; for example, including users of osseointegrated palatal implants in the non-users of OMIs group in the study by Bock and Ruf [5] could have skewed outcomes; and (5) poor reporting of a variety of issues (Tables 2, 3, 4, and 5) and negative critical appraisal scores for at least two appraisal criteria in all three included studies (Table 6) [35, 59]. To display these issues, we presented forests plots and the heterogeneity statistics of these three implementation constructs in Tables 10, 11, and 12 [64]. We used the fixed-effect model to display these plots because it better represents the relative weights of the individual studies than the random-effects model [86].

**Table 10:**
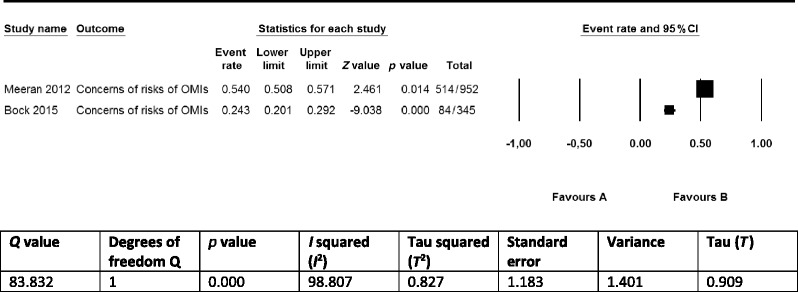
Prevalence of clinicians that are concerned regarding the risks of using OMIs

**Table 11:**
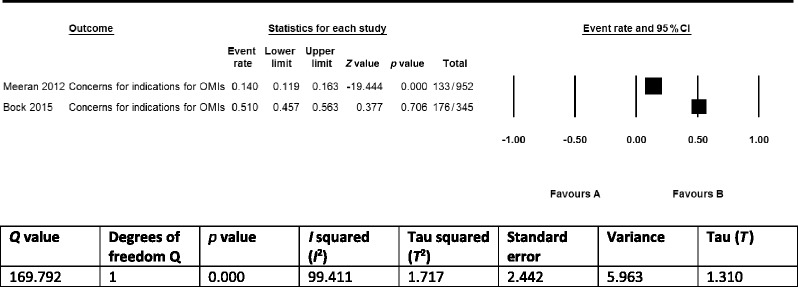
Prevalence of clinicians that are concerned with the limited indications for OMIs

**Table 12:**
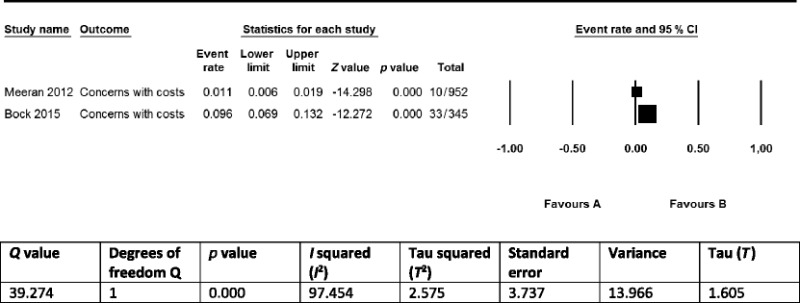
Prevalence of clinicians that are concerned with the costs of OMIs

The prevalence data on the use of OMIs by the 2784 clinicians in the studies by Meeran et al. [8] and Bock and Ruf [5] were not synthesized in a meta-analysis for most of the same reasons that were presented previously for implementation constructs (Table 13). Subgroup analyses and meta-regression were not conducted to explore heterogeneity or to address questions about specific stakeholders, interventions, or study designs because only two studies [5, 8] were included and data for the analysis of subgroups were not given by the authors of these studies [61].

**Table 13:**
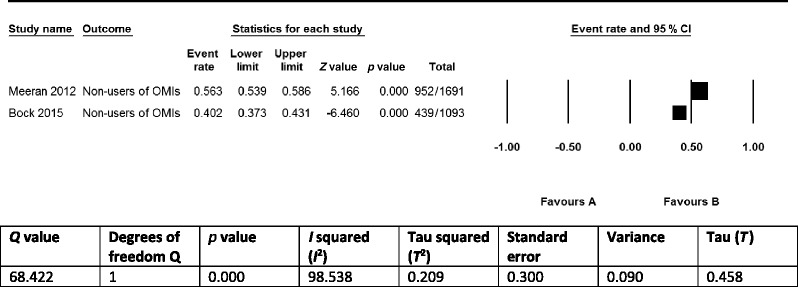
Prevalence of clinicians that do not use OMIs

### Qualitative synthesis and the robustness of the accumulative evidence

According to our protocol, we synthesized the findings exclusively in a narrative (qualitative) format because the criteria for conducting quantitative syntheses (see previous chapter) were not met for both the primary and secondary outcomes [14]. Seventeen constructs (with three overlapping) for the implementation of OMIs in clinical practice were identified in 3 eligible studies. These constructs were mostly self-explanatory but were not defined in any of these articles. One study [85] surveyed patients as stakeholders on potential barriers and facilitators to the use of OMIs. The high prevalence statistics of all four identified implementation constructs in this study demonstrated the importance of these items among patients (Table 8). A total of 13 barriers to the use of OMIs were identified for clinicians in two of the included studies [5, 8] (Table 8). Four of these barriers, i.e., (1) lack of training; (2) fear of risk factors like root damage and infection; (3) (almost) no suitable indications; and (4) skeptical about success/failure rates of OMIs, were scored by more than 50 % of the responding clinicians. The remaining nine barriers were scored by 33.3 % or less of these stakeholders. Three of the 13 barriers in these studies overlapped [5, 8] (Table 8). The prevalence statistics of these overlapping barriers varied widely between the two studies (Tables 10, 11, and 12).

The robustness of the accumulative evidence on these three implementation constructs was conditioned by: (1) the research design of the included studies (surveys); (2) the small number of eligible studies; (3) the limitations of the individual studies such as not defining or describing the research questions (the primary outcomes), using questionnaires with only closed-ended questions, not pilot testing of research procedures, using not-validated survey instruments (Tables 2, 3, 4, 5, 6, and 7 and Additional file 4); (4) the heterogeneity within and between studies such as differences in surveyed populations; and (5) the risk of reporting bias because protocols were not registered or published. These variables also downgraded the validity of the outcomes of these studies and significantly reduced their applicability to different contexts. The use of surveys as the research design and the exclusive use of non-defined closed-ended questions in these studies were key components for this downgrade.

The prevalence of clinicians that do not use OMIs in the two eligible studies that addressed our secondary research question was respectively 56.3 % (952/1691)[8] and 40.16 % (439/1093) [5]. The validity of these prevalence data was conditioned by many of the same limitations that were also identified for the primary research question.

### Contacting authors

Our proceedings for contacting authors and our questions for these stakeholders as well as their answers were presented in Additional file 5. Authors of two [5, 85] of the three included articles replied within 1 day to our “willingness to reply email” and subsequently addressed all our questions. Exchange of data was conducted in a positive and friendly context with both researchers. Their answers clarified various issues on their research studies and these issues were incorporated in Tables 2, 3, 4, 5, 6, 7, and 8. For example, Dr. Bock confirmed the numerators and denominators for the prevalence statistics of the implementation constructs. This information allowed us to quantify these constructs and to include them in forest plots (Tables 8, 9, 10, 11, and 12). The reference author and one of the co-authors of the third article [8] did not answer to our questions on their study (Additional file 5).

### Differences between protocol and review

All research proceedings in this systematic review were conducted according to our published protocol [14].

## Discussion

### Summary of main findings

No earlier systematic review had addressed our research questions. Three eligible studies were identified that addressed these questions. Prior to conducting this systematic review, three of the reviewers (RMR, LL, LR) created a list of potential barriers and facilitators to the implementation of OMIs in clinical practice. All 17 implementation constructs identified in the 3 eligible studies were also found in this list. These constructs were extracted from a total of 165 patients (Zawawi 2014) [85] and 1391 clinicians (952 (Meeran et al. 2012) [8] and 439 (Bock and Ruf 2015)[5]. Eight of the 17 identified barriers and facilitators were scored by more than 50 % of the pertinent stakeholders, which showed the importance of these constructs for these subjects (Table 8).

Our secondary research question was addressed by two of the eligible studies, which showed that respectively 56.3 % (952/1691) (Meeran et al. 2012) [8] and 40.16 % (439/1093) (Bock and Ruf 2015) [5] of clinicians do not use OMIs (Table 9). These high-prevalence statistics represented the knowledge-to-action gap and confirmed the need for asking our primary research question. These outcomes reflected the underuse of OMIs reported in several other studies [6, 7, 19] but were much better than those recorded by Banks et al. [4]. This latter survey was conducted in 2010 and found that 99.8 % (608/620) of British orthodontists do not use OMIs [4]. The validity and the applicability of the findings for our primary and secondary research questions were conditioned by a series of limitations that are discussed in the following sections.

### Limitations of the findings

The validity and the applicability of the findings of the primary research question of this systematic review should be considered in the context of the following variables: (1) the small number of eligible studies; (2) not defining or describing the research questions, i.e., the primary outcomes; (3) the research design (surveys) of the identified studies and the type of questions used; (4) the lack of pilot testing of research methods; (5) the various specific limitations within the selected studies (Tables 2, 3, 4, 5, 6, and 7 and Additional file 4); (6) heterogeneity between the studies; and (7) the risk of reporting bias.

The small number of identified eligible studies was an important limitation of the research findings of this systematic review. Verification of the research outcomes of the eligible studies was difficult because only one study [85] assessed implementation constructs for patients and just two studies [5, 8] assessed these items for clinicians. In addition, the questions for these pertinent stakeholders were not defined in any of these studies and mostly did not overlap. “The small number of eligible studies” issue is particularly important in the context of the numerous additional shortcomings of the selected studies (Tables 2, 3, 4, 5, 6, and 7 and Additional file 4).

All included studies used surveys with closed-ended questions as their research design, which impose important limitations: (1) feedback is only obtained on items that are explicitly asked about and (2) the risk of asking irrelevant and non-understandable questions [87]. These limitations particularly apply to the surveys of Meeran et al. [8] and Bock and Ruf [5].Other implementation constructs could have been more important than those surveyed in these studies because only 33.3 % (2/6) [8] and 28.6 % (2/7) [5] of barriers were scored by more than 50 % of the respondents. Poor understanding of survey questions was a major shortcoming of the study by Bock and Ruf [5]. Unclear definitions of OMIs and osseointegrated palatal implants had created misunderstanding among respondents in this study and could have flawed outcomes. Focus groups and in-depth interviews can be used to deal with these limitations of surveys. They can be conducted as pilot tests during the development of the survey questionnaires to identify possible implementation constructs and their importance. None of the included studies undertook such investigations nor pilot tested their research methods. In addition, none of these studies searched the literature to identify tested and validated standards for asking research questions on implementation constructs. Using new poorly defined questions is problematic when (1) interpreting and applying answers of respondents; (2) testing the reproducibility of answers; and (3) findings are included in a meta-analysis.

Numerous additional limitations were identified in the selected studies (Tables 2, 3, 4, 5, 6, and 7 and Additional file 4). Variables that could have influenced outcomes in Zawawi’s study [85] refer to including (1) respondents with and without prior knowledge on the interventional procedure with OMIs; (2) both patients and parents or siblings as respondents; and (3) respondents from different settings with possibly different characteristics (Table 7). Including patients that did and did not need OMIs in the survey was not an issue because these stakeholders were not informed about this treatment option prior to completing the questionnaires. This information and the fact that all patients were consecutively treated were obtained through contacting the authors (Additional file 5).

Outcomes in Meeran’s study [8] could be influenced by additional factors such as (1) the type of selection procedures of the 2100 enrolled orthodontists; (2) unclear reporting on response rates, i.e., numbers did not always add up (Tables 2, 4, and 9); and (3) urban respondents were overrepresented in the total sample and consisted of less non-users of OMIs than clinicians practicing in non-urban settings (Tables 2, 4, and 7 and Additional file 4). The importance of these issues could not be verified because both the author and a co-author did not reply to our questions (Additional file 5).

Additional variables that could have influenced outcomes in the study by Bock and Ruf [5] were (1) unclear terminology in the questionnaire; (2) including both strict non-users of OMIs and users of osseointegrated palatal implants as respondents on implementation constructs for OMIs. However, this latter subgroup of non-users consisted only of 5 % (22/439) of the total population of non-users of OMIs; (3) various types of experience with OMIs among the subgroups of non-users of OMIs; (4) the characteristics of the 439 non-users of OMIs was described but not those of the 345 respondents on implementation constructs. The rationale and the consequences of the dropout of the 94 non-responders was not discussed; and (5) various reporting issues (Tables 2, 5, 6, and 7 and Additional file 4).

Sources of heterogeneity between the selected studies included (1) the study design and size; (2) the characteristics of the respondents; (3) the type of interventions; (4) the setting and country; and (5) additional within study limitations that were outlined above. Heterogeneity was clearly depicted in the three forest plots of overlapping barriers to the implementation of OMIs and was confirmed by its statistics (Tables 10, 11, and 12). None of the studies had published or registered a protocol of their research study, which could have introduced reporting bias.

### Identifying and quantifying implementation constructs and dealing with them

The identification and quantification of barriers and facilitators to knowledge use are key for a successful knowledge translation plan [58, 88, 89]. High-prevalence statistics (>65 %) were identified for four implementation constructs for patients indicating their importance for these stakeholders [85] (Table 8). Patient-mediated implementation strategies can be developed to deal with these constructs. Such strategies could focus on informing patients and the wider public on the potential advantages of OMIs, for example, shorter treatment times, better treatment outcomes, less extractions, and that OMIs are the only options to obtain certain results [85]. Successful knowledge translation to these stakeholders could be obtained through: educational materials, financial incentives, consultations to explain the interventional procedure and its evidence base, videos of patients that have already undergone treatment with OMIs, and meetings with such stakeholders [89]. Informing patients on qualitative outcomes such as acceptance of the use of these devices and the associated pain and discomfort could be an important strategy to facilitate the implementation of OMIs [90–96]. In this context, it should be noted that Zawawi’s study [85] showed that the large majority, 91.6 % (76/83), of subjects that were surveyed after the insertion of OMIs recommended this treatment to others. This finding is congruent with other studies that assessed this issue [92, 95, 96].

Thirteen barriers to the use of OMIs were identified for clinicians in two of the selected studies [5, 8]. Three of these constructs overlapped (Tables 10, 11, and 12). Four barriers were scored by more than 50 % of the surveyed clinicians in these studies: (1) lack of training [8]; (2) fear of risk factors [8]; (3) (almost) no suitable indications [5]; and (4) skepticism about the success/failure rates of OMIs [5]. Strategies for dealing with these barriers could include printed educational materials, e.g., up-to-date syntheses and clinical practice guidelines [25], educational meetings, in particular small meetings with a particular topic [97], educational outreach, e.g., invitation of a clinical expert [98], opinion leaders [99], audit and feedback [100, 101], hands-on training, financial incentives, knowledge management interventions such as “evidence-based healthcare” training courses, peer meetings, and a variety of other tailored strategies [102]. Skepticism of clinicians about the success/failure rates of OMIs could also be the result of the low or moderate quality of research findings on OMIs that was identified in numerous systematic reviews and critical appraisals of articles on these devices [9, 11, 45, 46, 48, 103, 104]. Improving the quality of research studies on OMIs could be key in addressing his issue.

The effectiveness and efficiency of many of the presented knowledge translation interventions are not always well understood [89, 105, 106]. Choosing a knowledge translation intervention or a combination of them is both a “science” and an “art” [25, 107].

### Strengths and limitations of this systematic review

The strengths of this research study include (1) it was the first systematic review that addressed these research questions; (2) a protocol was submitted and published prior to applying the research methods of this systematic review, which reduced the risk of reporting bias [14]; (3) literature searches were conducted with broad-spectrum search strategies in a wide variety of databases and without language restrictions [53]; (4) data extraction tables were presented in great detail and were pilot tested; (5) this study was conducted independently by experienced systematic reviewers, methodologists, and topic experts, who had published several systematic reviews and review protocols on OMIs [14, 45–48]; (6) transparent reporting of all research proceedings in both the protocol and the final manuscript of this systematic review; and (7) authors were contacted by reviewers to obtain additional research data. These latter research procedures were used to verify reporting issues and to obtain additional research data. For example prevalence statistics of implementation constructs were clarified in the study by Bock and Ruf [5], which permitted the depicting of some of these statistics in forest plots and explore statistical heterogeneity (Tables 10, 11 and 12). The weaknesses of this systematic review were outlined previously in the section ‘Limitations of the findings’.

### Why these findings are important and for who

Notwithstanding the limitations of this systematic review, its findings are important because:

1. Seventeen barriers and facilitators were identified of which 8 were scored by more than 50 % of the surveyed stakeholders, demonstrating the importance of these implementation constructs.
2. The limitations and the small number of the eligible studies showed the need for additional studies on this research topic. The exclusive use of non-pilot tested, and non-defined closed-ended questions and not consulting potentially existing standards for exploring implementation constructs were key limitations of the included studies.
3. Of the high underuse of OMIs, which was confirmed by two [5, 8] of the selected studies which showed the severity of the knowledge-into-action gap. The problem of underuse of OMIs is probably more dramatic, because we only recorded the prevalence statistic of clinicians that do not use these devices. However, several forms of underuse of OMIs by clinicians exist: (1) never having used OMIs; (2) having used OMIs and stopped using them; and (3) using them infrequently. These three types of subgroups of underusers of OMIs should be considered separately in future research studies. For example Bock and Ruf [5] showed that only 1 % (4/417) of the users of OMIs used these devices on more than 2 patients per week, but most clinicians, i.e., 68.5 % (286/417), used OMIs infrequently (≤2 new patients/quarter). Underuse of OMIs among users was also recorded in other surveys [6, 18–20]. The underuse of OMIs was unexpected from a patient’s perspective, because most patients recommend interventions with OMIs to others [85, 92, 95, 96]. This underuse of OMIs was also unexpected from a clinician’s perspective, because of the numerous publications on their promising success rates, effectiveness, and wide applicability [9–11] and because they were included in the treatment plan for a common orthodontic patient by more than 75 % of 108 surveyed doctors [21]. Zawawi [85] indicated OMIs in 50.3 % (83/165) of consecutively treated patients.

The identification and quantification of the 17 implementation constructs and the high underuse of OMIs in clinical practice could be important rationales to redirect research studies of OMIs towards implementation issues. Wasting less research money and improving the quality of orthodontic treatment could be the consequence [108]. The findings of this systematic review are important for patients, clinicians, researchers, policymakers, insurance companies, implant companies, and research sponsors.

### What is next?

Future research should continue to focus on identifying and quantifying barriers and facilitators to the use of OMIs in clinical practice because this information is key to a successful knowledge translation plan [58, 88, 89]. The limitations of the included studies in this systematic review could be important items to consider when developing future studies. Conducting qualitative and quantitative systematic reviews of patient health experiences with OMIs is an essential initial step for evidence-based knowledge creation on implementation constructs. However, strategies to identify such constructs do not only include systematic reviews and surveys but also refer to (1) focus groups; (2) in-depth interviews; (3) talking to key individuals on the interventional procedures, e.g., clinical experts or guideline developers; (4) observing of the interventional procedure in action, e.g., in a clinical practice setting; and (5) brainstorming [25, 89]. These five methods can also be used as pilot tests or to fine-tune research questions for future survey questionnaires on implementation constructs. When such constructs are identified, tailored stakeholder-specific strategies can be developed to deal with them.

## Conclusions

This is the first systematic review that addressed implementation issues of OMI in clinical practice. The three eligible surveys [5, 8, 85] identified and quantified 17 implementation constructs. Three facilitators and one barrier (costs) were identified by more than 60 % of the orthodontic patients [85]. Lack of training, fear of risk factors, (almost) no suitable indications, and skepticism about additional benefit of OMIs were identified as barriers to the implementation of OMIs by more than 50 % of orthodontic clinicians [5, 8].

The main limitations of these studies included (1) the small number of studies; (2) not defining the research questions, i.e., the primary outcomes; (3) the research design (surveys) of the studies and the exclusive use of closed-ended questions; (4) not consulting standards for identifying implementation constructs; (5) the lack of pilot testing; (6) high heterogeneity; (7) the risk of reporting bias; and (8) additional shortcomings. This leaves much space for the exploration of additional constructs through open-ended questions in other research designs, such as in-depth interviews or focus groups. The severe underuse of OMIs that was identified in the selected studies demonstrated the need to identify and quantify such constructs and to develop strategies to deal with them.

Most primary studies on OMIs have addressed variables associated with their stability and effectiveness and few studies have assessed factors associated with their implementation. This study showed the need to change course. Before undertaking new research studies on OMIs, it will be necessary to consult the findings of this systematic review and other reviews and convene a variety of stakeholders, such as patients, clinicians, researchers, government bodies, guideline developers, and implant companies, to develop priority questions in this field of research [109]. Prioritizing such questions could redirect research on OMIs towards studies on their implementation. This could reduce additional research waste and benefit patients, clinicians, researchers, policymakers, insurance companies, implant companies, and research sponsors [108].

## Additional files

Additional file 1: PRISMA 2009 checklist. (DOC 63 kb)
Additional file 2: Records per data source. (DOCX 15 kb)
Additional file 3: Excluded full text articles. (DOCX 28 kb)
Additional file 4: Rationales for critical appraisal scores. (DOCX 30 kb)
Additional file 5: Questions for contacted authors and outcomes. (DOCX 28 kb)

## Abbreviations

OMIs: Orthodontic mini implants
