# Erratum to: Barriers and facilitators to the implementation of orthodontic mini implants in clinical practice: a systematic review

**DOI:** 10.1186/s13643-016-0359-5

**Published:** 2016-10-25

**Authors:** Reint Meursinge Reynders, Laura Ronchi, Luisa Ladu, Nicola Di Girolamo, Jan de Lange, Nia Roberts, Sharon Mickan

**Affiliations:** 1 Department of Oral and Maxillofacial Surgery, Academic Medical Center, University of Amsterdam, Meibergdreef 9, 1105 AZ Amsterdam, The Netherlands; 2 Via Matteo Bandello 15, 20123 Milan, Italy; 3 Department of Veterinary Sciences, University of Bologna, Via Tolara di Sopra 50, 40064 Ozzano dell’Emilia, BO Italy; 4 Department of Oral and Maxillofacial Surgery, Academic Medical Center and Academisch Centrum Tandheelkunde Amsterdam (ACTA), University of Amsterdam, Meibergdreef 9, 1105 AZ Amsterdam, The Netherlands; 5 Bodleian Health Care libraries, Cairns Library Level 3, John Radcliffe Hospital, University of Oxford, Oxford, OX3 9DU UK; 6 Department of Allied Health, Gold Coast Health and Griffith University, Queensland, QLD 4222 Australia

## Erratum

After publication of the original article [1], the authors noticed that one of their correction requests had been missed:

In Table 8, column “Prevalence of the barrier”, a superscripted “a” was included next to the “5 %” and “1 %” prevalence data. These “a”s should have been removed. This is now corrected in the original article and also shown in the below table. We apologise for any inconvenience caused by this error.

**Table 8:**
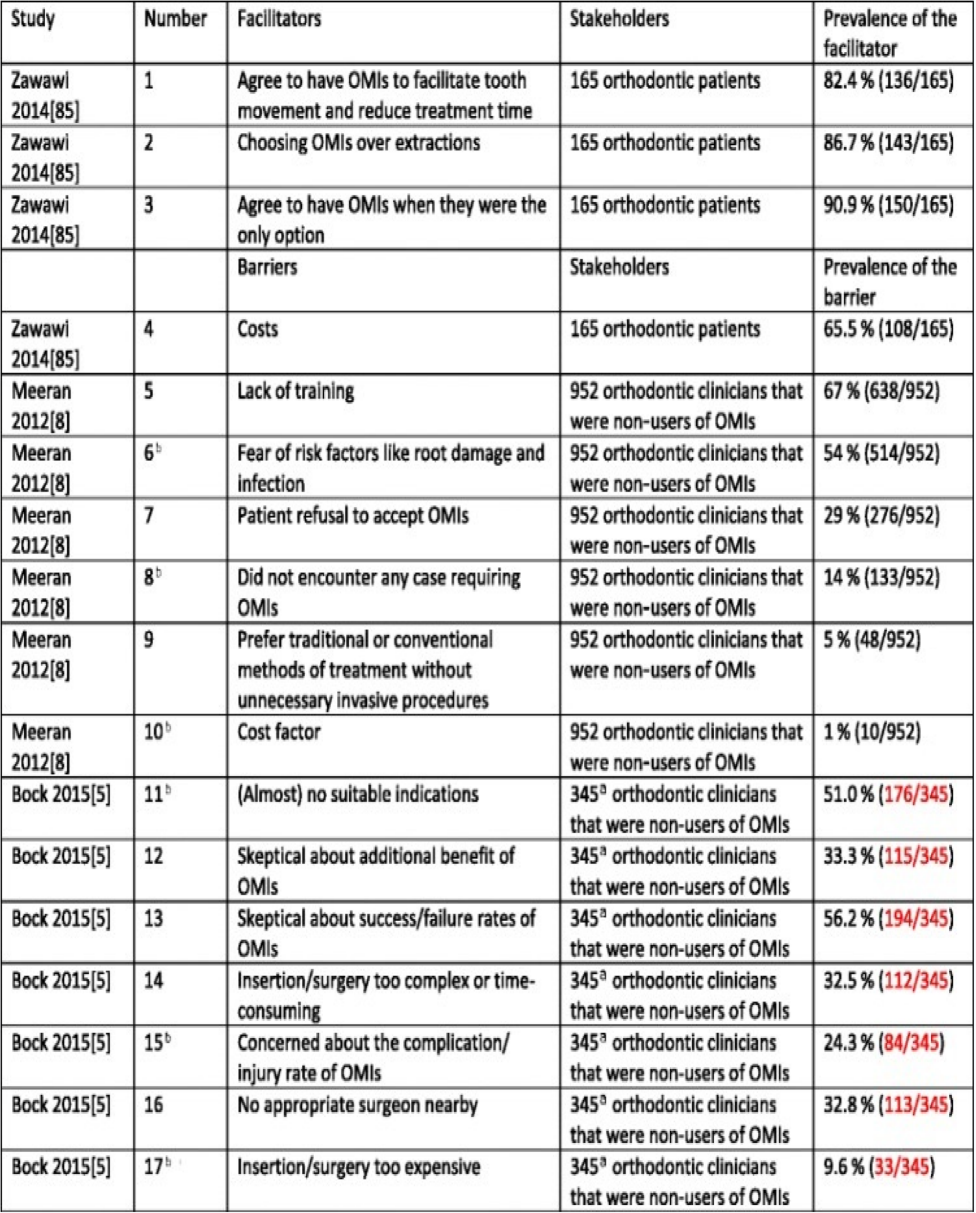
Barriers and facilitators to the implementation of OMIs^a^

Items in black-type face represent those of the published manuscript. Items in  represent those obtained through contacting the authors of the pertinent manuscript

^a^The numerators and denominators were not completely clear in the published article and were confirmed through contacting the authors of this research study

^b^Barriers 6 and 15, 8 and 11, and 10 and 17 overlap
