# Supplementary material for: Barriers and facilitators to the implementation of orthodontic mini implants in clinical practice: a systematic review
Source: Syst Rev. 2016 Sep 23;5:163. doi: 10.1186/s13643-016-0336-z (PMC5034676; doi:10.1186/s13643-016-0336-z)
Supplement: Additional file 2: — Records per data source. (DOCX 15 kb) [file 13643_2016_336_MOESM2_ESM.docx]

**Additional file 2. Records per data source**

**Records retrieved by electronic searching, hand searching, and reference searching * ****

| **Source of records** | **Date of search** | **Number of abstracts** |
| --- | --- | --- |
| Google Scholar Beta | January 15 2016 | 6290 |
| PubMed (MEDLINE) | January 15 2016 | 2020 |
| EMBASE (Ovid) | Week 2 2016 | 2807 |
| Cochrane Central Register of Controlled Trials (CENTRAL) | January 15 2016 | 36 |
| “Related Articles” in PubMed | January 15 2016 | 142 |
| CINAHL | January 15 2016 | 612 |
| PsycINFO | January 15 2016 | 75 |
| PROSPERO | January 15 2016 | 181 |
| Web of Science | January 15 2016 | 1776 |
| Sociological Abstracts | January 15 2016 | 13 |
| African Index Medicus | January 15 2016 | 6 |
| African Journals online (AJOL) | January 15 2016 | 2 |
| Informit Health Collection | January 15 2016 | 24 |
| Index Medicus for the Eastern Mediterranean Region | January 15 2016 | 0 |
| IndMED | January 15 2016 | 280 |
| KoreaMed | January 15 2016 | 916 |
| LILACS | January 15 2016 | 87 |
| Index Medicus for the South-East Asia Region (IMSEAR) | January 15 2016 | 0 |
| Western Pacific Region Index Medicus (WPRIM) | January 15 2016 | 1218 |
| Open Grey | January 15 2016 | 114 |
| The National Technical Information Service (NTIS) | January 15 2016 | 20 |
| The Health Management Information Consortium (HMIC) | January 15 2016 | 2 |
| ProQuest Dissertations & Theses | January 15 2016 | 4 |
| Meeting Abstracts | January 15 2016 | 5 |
| ISI Proceedings | January 15 2016 | 0 |
| IEEE Conference proceedings | January 15 2016 | 8 |
| Database of Abstracts of Reviews of Effects (DARE) in the Centre for Reviews and Dissemination (CRD) Database | January 15 2016 | 199 |
| Health Technology Assessment database (HTA) in the Centre for Reviews and Dissemination (CRD) Database | January 15 2016 | 15 |
| NHS EED | January 15 2016 | 15 |
| Turning research into practice (TRIP) Database | January 15 2016 | 850 |
| SUMSearch2 | January 15 2016 | 53 |
| MedlinePlus Guidelines | January 15 2016 | 106 |
| Guidelines of the Australian National Health and Medical Research Council | January 15 2016 | 6 |
| Guidelines of the Canadian Medical Association | January 15 2016 | 0 |
| National Guideline Clearinghouse | January 15 2016 | 8 |
| National Library of Medicine Guidelines | January 15 2016 | 1 |
| New Zealand Guidelines Group | January 15 2016 | 3 |
| NICE Clinical Guidelines | January 15 2016 | 6 |
| Citation alerts in Pubmed and Ovid | January 15 2016 | 88 |
| Handsearching | January 15 2016 | 3 |
| Reference lists | January 15 2016 | 28 |
| Correspondence | January 15 2016 | 2 |
|  |  |  |
| **Total** |  | 18021 Abstracts with overlap |

* All search strategies were copied and pasted from the original search strategy without re-typing,

because this procedure can introduce errors [53]. To avoid the inappropriate exclusion

of pertinent articles, broader searches were conducted when no abstracts were found with the

reference search strategy, i.e., the search strategy used for Pubmed (MEDLINE).

** For each individual search engine we applied the appropriate characters to truncate or explore

search terms.
