# Supplementary material for: Barriers and facilitators to the implementation of orthodontic mini implants in clinical practice: a systematic review
Source: Syst Rev. 2016 Sep 23;5:163. doi: 10.1186/s13643-016-0336-z (PMC5034676; doi:10.1186/s13643-016-0336-z)
Supplement: Additional file 3: — Excluded full text articles. (DOCX 28 kb) [file 13643_2016_336_MOESM3_ESM.docx]

**Additional file 3. Excluded full-text articles and references**

**Excluded full-text articles and reasons for exclusion (n=34)**

| **Author(s)** | **Reasons for exclusion*** |
| --- | --- |
| Acar 2015 | A |
| Anwar 2010 | B |
| Asscherickx 2010 | A |
| Banks 2010 | A |
| Barthelemi 2015 | A |
| Baxmann 2010 | B |
| Buschang 2008 | A |
| Bustamante Capucho Brandão 2008 | B |
| Chen 2011 | B |
| Cornelis 2008 | B |
| Dwijendra 2015 | B |
| Feldmann 2007 | C |
| Feldmann 2012 | C |
| Garfinkle 2008 | B |
| Guimarães Blaya 2010 | B |
| Gündüz 2004 | B |
| Hu 2010 | B |
| Hyde 2010 | A |
| Justens 2008 | B |
| Kawaguchi 2014 | B |
| Keim 2008 | A |
| Keim 2014 | A |
| Kuroda 2007 | B |
| Lamberton 2016 | B |
| Lee 2008 | B |
| Lehnen 2011a | B |
| Lehnen 2011b | B |
| Markic 2014 | A |
| Patil 2012 | A |
| Pithon 2015 | B |
| Rampon 2013 | A |
| Sandhu 2013 | B |
| Shirck 2009 | A |
| Tseng 2010 | B |

***Reasons for exclusion**

A: The use of orthodontic mini-implants was recorded, but not implementation constructs

B: Patient health experiences were recorded, but not implementation constructs

C: Patient health experiences were recorded, but not implementation constructs. Interventional techniques were also not all eligible.

**References excluded full-text articles**

**Acar 2015**

Acar YB, Hergel CA, Ateş M, Küçükkeleş N. Mini-implant Usage in Orthodontic Practice. Turkish Journal of Orthodontics: March 2015, Vol. 28, No. 1, pp. 1-6.

**Anwar 2010**

Anwar A, Raja UB, Naureen S. Success rate and pain perception in orthodontic patients receiving bone screws for anchorage: A pilot study. Pakistan Oral & Dent J 2010;30(1):101-103.

**Asscherickx 2010**

[Asscherickx K](http://www.ncbi.nlm.nih.gov/pubmed/?term=Asscherickx%20K%5BAuthor%5D&cauthor=true&cauthor_uid=20122439), [Vannet BV](http://www.ncbi.nlm.nih.gov/pubmed/?term=Vannet%20BV%5BAuthor%5D&cauthor=true&cauthor_uid=20122439), [Bottenberg P](http://www.ncbi.nlm.nih.gov/pubmed/?term=Bottenberg%20P%5BAuthor%5D&cauthor=true&cauthor_uid=20122439), [Wehrbein H](http://www.ncbi.nlm.nih.gov/pubmed/?term=Wehrbein%20H%5BAuthor%5D&cauthor=true&cauthor_uid=20122439), [Sabzevar MM](http://www.ncbi.nlm.nih.gov/pubmed/?term=Sabzevar%20MM%5BAuthor%5D&cauthor=true&cauthor_uid=20122439). Clinical observations and success rates of palatal implants. [Am J Orthod Dentofacial Orthop.](http://www.ncbi.nlm.nih.gov/pubmed/?term=clinical+observations+and+succcess+rates+of+palatal+implants) 2010 Jan;137(1):114-22.

**Banks 2010**

Banks P, Elton V, Jones Y, Rice P, Denwent S, Odondi L. The use of fixed appliances in the UK: a survey of specialist orthodontics. J Orthod 2010;37(1):43-55.

**Barthelemi 2015**

Barthelemi S, Beauval H. [Prevalence of the use of anchorage miniscrews among French orthodontists.](http://www.ncbi.nlm.nih.gov/pubmed/26507966)

Int Orthod. 2015 Dec;13(4):436-61.

**Baxmann 2010**

[Baxmann M](http://www.ncbi.nlm.nih.gov/pubmed?term=Baxmann%20M%5BAuthor%5D&cauthor=true&cauthor_uid=20816284), [McDonald F](http://www.ncbi.nlm.nih.gov/pubmed?term=McDonald%20F%5BAuthor%5D&cauthor=true&cauthor_uid=20816284), [Bourauel C](http://www.ncbi.nlm.nih.gov/pubmed?term=Bourauel%20C%5BAuthor%5D&cauthor=true&cauthor_uid=20816284), [Jäger A](http://www.ncbi.nlm.nih.gov/pubmed?term=J%C3%A4ger%20A%5BAuthor%5D&cauthor=true&cauthor_uid=20816284). Expectations, acceptance, and preferences regarding microimplant treatment in orthodontic patients: A randomized controlled trial. [Am J Orthod Dentofacial Orthop.](http://www.ncbi.nlm.nih.gov/pubmed/20816284) 2010 Sep;138(3):250.e1-250.e10; discussion 250-1.

**Buschang 2008**

[Buschang PH](http://www.ncbi.nlm.nih.gov/pubmed?term=Buschang%20PH%5BAuthor%5D&cauthor=true&cauthor_uid=18974458), [Carrillo R](http://www.ncbi.nlm.nih.gov/pubmed?term=Carrillo%20R%5BAuthor%5D&cauthor=true&cauthor_uid=18974458), [Ozenbaugh B](http://www.ncbi.nlm.nih.gov/pubmed?term=Ozenbaugh%20B%5BAuthor%5D&cauthor=true&cauthor_uid=18974458), [Rossouw PE](http://www.ncbi.nlm.nih.gov/pubmed?term=Rossouw%20PE%5BAuthor%5D&cauthor=true&cauthor_uid=18974458). 2008 survey of AAO members on miniscrew usage. [J Clin Orthod.](http://www.ncbi.nlm.nih.gov/pubmed/18974458) 2008;42(9):513-8.

**Bustamante Capucho Brandão 2008**

Bustamante Capucho Brandão L, Nelson Mucha J. Rate of mini-implant acceptance by patients undergoing orthodontic treatment – A preliminary study with questionnaires. Dental Press J. Orthod. 2008;13(5):118-127.

**Chen 2011**

Chen CM, Chang CS, Tseng YC, Hsu KR, Lee KT, Lee HE. The perception of pain following interdental microimplant treatment for skeletal anchorage: a retrospective study. Odontology 2011 Jan;99(1):88-91.

**Cornelis 2008**

[Cornelis MA](http://www.ncbi.nlm.nih.gov/pubmed?term=Cornelis%20MA%5BAuthor%5D&cauthor=true&cauthor_uid=18174066), [Scheffler NR](http://www.ncbi.nlm.nih.gov/pubmed?term=Scheffler%20NR%5BAuthor%5D&cauthor=true&cauthor_uid=18174066), [Nyssen-Behets C](http://www.ncbi.nlm.nih.gov/pubmed?term=Nyssen-Behets%20C%5BAuthor%5D&cauthor=true&cauthor_uid=18174066), [De Clerck HJ](http://www.ncbi.nlm.nih.gov/pubmed?term=De%20Clerck%20HJ%5BAuthor%5D&cauthor=true&cauthor_uid=18174066), [Tulloch JF](http://www.ncbi.nlm.nih.gov/pubmed?term=Tulloch%20JF%5BAuthor%5D&cauthor=true&cauthor_uid=18174066). Patients' and orthodontists' perceptions of miniplates used for temporary skeletal anchorage: a prospective study. [Am J Orthod Dentofacial Orthop.](http://www.ncbi.nlm.nih.gov/pubmed/?term=cornelis+ma+patients+and+orthodontists+perceptions) 2008 Jan;133(1):18-24.

**Dwijendra 2015**

Dwijendra KS, Gheware A, Patil SK, Inchanalkar R, Gugwad S, Kathariya MD. Experience of Pediatric Patients with Mini-Implants undergoing Orthodontic Treatment. Journal of International Oral Health 2015; 7(10):112-115.

**Feldmann 2007**

Feldmann I, List T, Feldmann H, Bondemark L. [Pain intensity and discomfort following surgical placement of orthodontic anchoring units and premolar extraction: a randomized controlled trial.](http://www.ncbi.nlm.nih.gov/pubmed/17605489) Angle Orthod. 2007 Jul;77(4):578-85.

**Feldmann 2012**

[Feldmann I](http://www.ncbi.nlm.nih.gov/pubmed?term=Feldmann%20I%5BAuthor%5D&cauthor=true&cauthor_uid=21300723), [List T](http://www.ncbi.nlm.nih.gov/pubmed?term=List%20T%5BAuthor%5D&cauthor=true&cauthor_uid=21300723), [Bondemark L](http://www.ncbi.nlm.nih.gov/pubmed?term=Bondemark%20L%5BAuthor%5D&cauthor=true&cauthor_uid=21300723). Orthodontic anchoring techniques and its influence on pain, discomfort, and jaw function--a randomized controlled trial. [Eur J Orthod.](http://www.ncbi.nlm.nih.gov/pubmed/21300723) 2012 Feb;34(1):102-8.

**Garfinkle 2008**

[Garfinkle JS](http://www.ncbi.nlm.nih.gov/pubmed?term=Garfinkle%20JS%5BAuthor%5D&cauthor=true&cauthor_uid=18456137), [Cunningham LL Jr](http://www.ncbi.nlm.nih.gov/pubmed?term=Cunningham%20LL%20Jr%5BAuthor%5D&cauthor=true&cauthor_uid=18456137), [Beeman CS](http://www.ncbi.nlm.nih.gov/pubmed?term=Beeman%20CS%5BAuthor%5D&cauthor=true&cauthor_uid=18456137), [Kluemper GT](http://www.ncbi.nlm.nih.gov/pubmed?term=Kluemper%20GT%5BAuthor%5D&cauthor=true&cauthor_uid=18456137), [Hicks EP](http://www.ncbi.nlm.nih.gov/pubmed?term=Hicks%20EP%5BAuthor%5D&cauthor=true&cauthor_uid=18456137), [Kim MO](http://www.ncbi.nlm.nih.gov/pubmed?term=Kim%20MO%5BAuthor%5D&cauthor=true&cauthor_uid=18456137). Evaluation of orthodontic mini-implant anchorage in premolar extraction therapy in adolescents. [Am J Orthod Dentofacial Orthop.](http://www.ncbi.nlm.nih.gov/pubmed/18456137) 2008 May;133(5):642-53.

**Guimarães Blaya 2010**

Guimarães Blaya M, Segatto Blaya D, Beck Guimarães M, Hirakata LM, Marquezan M. Patient’s perception on mini-screws used formolar distalization. Rev. odonto ciênc. 2010;25(3):266-270

**Gündüz**

Gündüz E, Schneider-Del Savio TT, Kucher G, Schneider B, Bantleon HP. [Acceptance rate of palatal implants: a questionnaire study.](http://www.ncbi.nlm.nih.gov/pubmed/15520697) Am J Orthod Dentofacial Orthop. 2004 Nov;126(5):623-6.

**Hu 2010**

[Hu QW](http://www.ncbi.nlm.nih.gov/pubmed/?term=Hu%20QW%5BAuthor%5D&cauthor=true&cauthor_uid=21431256), [Tao L](http://www.ncbi.nlm.nih.gov/pubmed/?term=Tao%20L%5BAuthor%5D&cauthor=true&cauthor_uid=21431256), [Zhao N](http://www.ncbi.nlm.nih.gov/pubmed/?term=Zhao%20N%5BAuthor%5D&cauthor=true&cauthor_uid=21431256). Acceptance rate of miniscrew application during orthodontic treatment. [Shanghai Kou Qiang Yi Xue.](http://www.ncbi.nlm.nih.gov/pubmed/21431256) 2010 Dec;19(6):590-3.

**Hyde 2010**

[Hyde JD](http://www.ncbi.nlm.nih.gov/pubmed?term=Hyde%20JD%5BAuthor%5D&cauthor=true&cauthor_uid=21105585), [King GJ](http://www.ncbi.nlm.nih.gov/pubmed?term=King%20GJ%5BAuthor%5D&cauthor=true&cauthor_uid=21105585), [Greenlee GM](http://www.ncbi.nlm.nih.gov/pubmed?term=Greenlee%20GM%5BAuthor%5D&cauthor=true&cauthor_uid=21105585), [Spiekerman C](http://www.ncbi.nlm.nih.gov/pubmed?term=Spiekerman%20C%5BAuthor%5D&cauthor=true&cauthor_uid=21105585), [Huang GJ](http://www.ncbi.nlm.nih.gov/pubmed?term=Huang%20GJ%5BAuthor%5D&cauthor=true&cauthor_uid=21105585). Survey of orthodontists' attitudes and experiences regarding miniscrew implants. [J Clin Orthod.](http://www.ncbi.nlm.nih.gov/pubmed/?term=Survey+of+Orthodontists%E2%80%99+Attitudes+and+Experiences+Regarding+Miniscrew+Implants) 2010;44(8):481-6.

**Justens 2008**

[Justens E](http://www.ncbi.nlm.nih.gov/pubmed?term=Justens%20E%5BAuthor%5D&cauthor=true&cauthor_uid=18384412), [De Bruyn H](http://www.ncbi.nlm.nih.gov/pubmed?term=De%20Bruyn%20H%5BAuthor%5D&cauthor=true&cauthor_uid=18384412). Clinical outcome of mini-screws used as orthodontic anchorage. [Clin Implant Dent Relat Res.](http://www.ncbi.nlm.nih.gov/pubmed/?term=clinical+outcome+of+mini-screws+used+justens) 2008 Sep;10(3):174-80.

**Kawaguchi 2014**

Kawaguchi M, Miyazawa K, Tabuchi M, Fuyamada M, Goto S. Questionnaire survey on pain and discomfort after insertion of orthodontic buccal miniscrews, palatal miniscrews and, orthodontic miniplates. Orthod Waves 2014;73:1-7.

**Keim 2008**

[Keim RG](http://www.ncbi.nlm.nih.gov/pubmed/?term=Keim%20RG%5BAuthor%5D&cauthor=true&cauthor_uid=25416338), [Gottlieb EL](http://www.ncbi.nlm.nih.gov/pubmed/?term=Gottlieb%20EL%5BAuthor%5D&cauthor=true&cauthor_uid=25416338), Nelson A, [Vogels DS 3rd](http://www.ncbi.nlm.nih.gov/pubmed/?term=Vogels%20DS%203rd%5BAuthor%5D&cauthor=true&cauthor_uid=25416338),: 2008 JCO study of orthodontic diagnosis and treatment procedures, Part 1: results and trends. J Clin Orthod 2008, 42(11):625-640.

**Keim 2014**

[Keim RG](http://www.ncbi.nlm.nih.gov/pubmed/?term=Keim%20RG%5BAuthor%5D&cauthor=true&cauthor_uid=25416338), [Gottlieb EL](http://www.ncbi.nlm.nih.gov/pubmed/?term=Gottlieb%20EL%5BAuthor%5D&cauthor=true&cauthor_uid=25416338), [Vogels DS 3rd](http://www.ncbi.nlm.nih.gov/pubmed/?term=Vogels%20DS%203rd%5BAuthor%5D&cauthor=true&cauthor_uid=25416338), [Vogels PB](http://www.ncbi.nlm.nih.gov/pubmed/?term=Vogels%20PB%5BAuthor%5D&cauthor=true&cauthor_uid=25416338): 2014 JCO study of orthodontic diagnosis and treatment procedures, Part 1: results and trends. J Clin Orthod 2014, 48(10):607-30.

**Kuroda 2007**

[Kuroda S](http://www.ncbi.nlm.nih.gov/pubmed?term=Kuroda%20S%5BAuthor%5D&cauthor=true&cauthor_uid=17208101), [Sugawara Y](http://www.ncbi.nlm.nih.gov/pubmed?term=Sugawara%20Y%5BAuthor%5D&cauthor=true&cauthor_uid=17208101), [Deguchi T](http://www.ncbi.nlm.nih.gov/pubmed?term=Deguchi%20T%5BAuthor%5D&cauthor=true&cauthor_uid=17208101), [Kyung HM](http://www.ncbi.nlm.nih.gov/pubmed?term=Kyung%20HM%5BAuthor%5D&cauthor=true&cauthor_uid=17208101), [Takano-Yamamoto T](http://www.ncbi.nlm.nih.gov/pubmed?term=Takano-Yamamoto%20T%5BAuthor%5D&cauthor=true&cauthor_uid=17208101). Clinical use of miniscrew implants as orthodontic anchorage: success rates and postoperative discomfort. [Am J Orthod Dentofacial Orthop.](http://www.ncbi.nlm.nih.gov/pubmed/?term=kuroda++clinical+use+of+miniscrew+implants) 2007 Jan;131(1):9-15.

**Lamberton 2016**

[Lamberton JA](http://www.ncbi.nlm.nih.gov/pubmed/?term=Lamberton%20JA%5BAuthor%5D&cauthor=true&cauthor_uid=26718373), [Oesterle LJ](http://www.ncbi.nlm.nih.gov/pubmed/?term=Oesterle%20LJ%5BAuthor%5D&cauthor=true&cauthor_uid=26718373), [Shellhart WC](http://www.ncbi.nlm.nih.gov/pubmed/?term=Shellhart%20WC%5BAuthor%5D&cauthor=true&cauthor_uid=26718373), [Newman SM](http://www.ncbi.nlm.nih.gov/pubmed/?term=Newman%20SM%5BAuthor%5D&cauthor=true&cauthor_uid=26718373), [Harrell RE](http://www.ncbi.nlm.nih.gov/pubmed/?term=Harrell%20RE%5BAuthor%5D&cauthor=true&cauthor_uid=26718373), [Tilliss T](http://www.ncbi.nlm.nih.gov/pubmed/?term=Tilliss%20T%5BAuthor%5D&cauthor=true&cauthor_uid=26718373), [Singh N](http://www.ncbi.nlm.nih.gov/pubmed/?term=Singh%20N%5BAuthor%5D&cauthor=true&cauthor_uid=26718373), [Carey CM](http://www.ncbi.nlm.nih.gov/pubmed/?term=Carey%20CM%5BAuthor%5D&cauthor=true&cauthor_uid=26718373). Comparison of pain perception during miniscrew placement in orthodontic patients with a visual analog scale survey between compound topical and needle-injected anesthetics: A crossover, prospective, randomized clinical trial. [Am J Orthod Dentofacial Orthop.](http://www.ncbi.nlm.nih.gov/pubmed/?term=Comparison+of+pain+perception+during+miniscrew+placement+in+orthodontic+patients+with+a+visual+analog+scale+survey+between+compound+topical+and+needle-injected+anesthetics%3A+A+crossover%2C+prospective%2C+randomized+clinical+trial) 2016 Jan;149(1):15-23.

**Lee 2008**

Lee TC, McGrath CP, Wong RW, Rabie AB. [Patients' perceptions regarding microimplant as anchorage in orthodontics.](http://www.ncbi.nlm.nih.gov/pubmed/18251610) Angle Orthod. 2008 Mar;78(2):228-33.

**Lehnen 2011a**

[Lehnen S](http://www.ncbi.nlm.nih.gov/pubmed?term=Lehnen%20S%5BAuthor%5D&cauthor=true&cauthor_uid=21503849), [McDonald F](http://www.ncbi.nlm.nih.gov/pubmed?term=McDonald%20F%5BAuthor%5D&cauthor=true&cauthor_uid=21503849), [Bourauel C](http://www.ncbi.nlm.nih.gov/pubmed?term=Bourauel%20C%5BAuthor%5D&cauthor=true&cauthor_uid=21503849), [Baxmann M](http://www.ncbi.nlm.nih.gov/pubmed?term=Baxmann%20M%5BAuthor%5D&cauthor=true&cauthor_uid=21503849). Patient expectations, acceptance and preferences in treatment with orthodontic mini-implants. A randomly controlled study. Part I: insertion techniques. [J Orofac Orthop.](http://www.ncbi.nlm.nih.gov/pubmed/21503849) 2011 Mar;72(2):93-102.

**Lehnen 2011b**

[Lehnen S](http://www.ncbi.nlm.nih.gov/pubmed?term=Lehnen%20S%5BAuthor%5D&cauthor=true&cauthor_uid=21744200), [McDonald F](http://www.ncbi.nlm.nih.gov/pubmed?term=McDonald%20F%5BAuthor%5D&cauthor=true&cauthor_uid=21744200), [Bourauel C](http://www.ncbi.nlm.nih.gov/pubmed?term=Bourauel%20C%5BAuthor%5D&cauthor=true&cauthor_uid=21744200), [Jäger A](http://www.ncbi.nlm.nih.gov/pubmed?term=J%C3%A4ger%20A%5BAuthor%5D&cauthor=true&cauthor_uid=21744200), [Baxmann M](http://www.ncbi.nlm.nih.gov/pubmed?term=Baxmann%20M%5BAuthor%5D&cauthor=true&cauthor_uid=21744200). Expectations, acceptance and preferences of patients in treatment with orthodontic mini-implants: part II: implant removal. [J Orofac Orthop.](http://www.ncbi.nlm.nih.gov/pubmed/21744200) 2011 Jul;72(3):214-22.

**Markic 2014**

[Markic G](http://www.ncbi.nlm.nih.gov/pubmed?term=Markic%20G%5BAuthor%5D&cauthor=true&cauthor_uid=24935644), [Katsaros C](http://www.ncbi.nlm.nih.gov/pubmed?term=Katsaros%20C%5BAuthor%5D&cauthor=true&cauthor_uid=24935644), [Pandis N](http://www.ncbi.nlm.nih.gov/pubmed?term=Pandis%20N%5BAuthor%5D&cauthor=true&cauthor_uid=24935644), [Eliades T](http://www.ncbi.nlm.nih.gov/pubmed?term=Eliades%20T%5BAuthor%5D&cauthor=true&cauthor_uid=24935644). Temporary anchorage device usage: a survey among Swiss orthodontists. Prog Orthod 2014 Apr 1;15(1):29.

**Patil 2012**

Patil A, Revankar AV. Evaluation of microimplant use in orthodontic practices in India: An opinion-based survey. J Ind Orthod Soc 2012; 46:269-272.

**Pithon 2015**

Pithon MM, Santos MJ, Ribeiro MC, Nascimento RC, Rodrigues RS, Ruellas AC, Coqueiro RS. Patients’ perceptions of installation, use and results of orthodontic mini—implants. Acta Odontol. Latinoam. 2015;28(2):108-112.

**Rampon 2013**

[Rampon FB](http://www.ncbi.nlm.nih.gov/pubmed/?term=Rampon%20FB%5BAuthor%5D&cauthor=true&cauthor_uid=23876966), [Nóbrega C](http://www.ncbi.nlm.nih.gov/pubmed/?term=N%C3%B3brega%20C%5BAuthor%5D&cauthor=true&cauthor_uid=23876966), [Bretos JL](http://www.ncbi.nlm.nih.gov/pubmed/?term=Bretos%20JL%5BAuthor%5D&cauthor=true&cauthor_uid=23876966), [Arsati F](http://www.ncbi.nlm.nih.gov/pubmed/?term=Arsati%20F%5BAuthor%5D&cauthor=true&cauthor_uid=23876966), [Jakob S](http://www.ncbi.nlm.nih.gov/pubmed/?term=Jakob%20S%5BAuthor%5D&cauthor=true&cauthor_uid=23876966), [Jimenez-Pellegrin MC](http://www.ncbi.nlm.nih.gov/pubmed/?term=Jimenez-Pellegrin%20MC%5BAuthor%5D&cauthor=true&cauthor_uid=23876966). Profile of the orthodontist practicing in the State of São Paulo--part 2. [Dental Press J Orthod.](http://www.ncbi.nlm.nih.gov/pubmed/?term=Profile+of+the+orthodontist+practicing+in+the+State+of+S%C3%A3o+Paulo) 2013 Feb 15;18(1):32.e1-6.

**Sandhu 2013**

Sandhu JS, Sandhu SV, Bector K, Sandhu SS. Patients' perception and postoperative discomfort with mini-implants. J Ind Orthod Soc 2013;47(4):199-201.

**Shirck 2009**

Shirck JM. Survey of temporary anchorage device utilization in graduate orthodontic programs and orthodontic practices in the United States. MSc thesis. Ohio (USA). The Ohio State University, Department of Dentistry; 2009.

**Tseng 2010**

Tseng YC, Chen CM, Wang HC, Wang CH, Lee HE, Lee KT. Pain perception during miniplate-assisted orthodontic therapy. Kaohsiung J Med Sci. 2010 Nov;26(11):603-8.
