# Supplementary material for: Barriers and facilitators to the implementation of orthodontic mini implants in clinical practice: a systematic review
Source: Syst Rev. 2016 Sep 23;5:163. doi: 10.1186/s13643-016-0336-z (PMC5034676; doi:10.1186/s13643-016-0336-z)
Supplement: Additional file 4: — Rationales for critical appraisal scores. (DOCX 30 kb) [file 13643_2016_336_MOESM4_ESM.docx]

**Additional file 4. Rationales for critical appraisal scores**

**Table 1. The Joanna Briggs Institute critical appraisal tool [35,59] of prevalence and incidence data for the study by Zawawi (2014)[85]***

| **Appraisal questions** | **Appraisal scores** | **Comments** |
| --- | --- | --- |
| 1)Was the sample representative of the target population ? | ☺ | Patients that were seeking orthodontic treatment were included. |
| 2)Were study participants recruited in an appropriate way ? | ☺ | Not much information was provided by the author on the selection procedures. However, the author was contacted and explained that the recruited patients were consecutively treated. This domain was subsequently upgraded from ‘Unclear’ to ‘Yes’. |
| 3)Was the sample size adequate ? | ☺ | Sample size was adequate |
| 4)Were the study subjects and the setting described in detail ? | ? | Numbers and demographics of patients in the respective settings were not reported |
| 5)Was the data analysis conducted with sufficient coverage of the identified sample ? | ☺ | All stakeholders responded |
| 6)Were objective, standard criteria used for the measurement of the condition ? | ☹ | No reference was made to a reference standard for assessing implementation constructs. Survey instruments were not validated. |
| 7)Was the condition measured reliably ? | ☺ | Yes, only closed-ended questions |
| 8)Was there appropriate statistical analysis ? | ☺ | Yes, only prevalence data were presented with appropriate numerators and denominators |
| 9)Are all important confounding factors/subgroups/  differences identified and accounted for ? | ☹ | Confounding factors/subgroups included:  1)non-defined, not-pilot-tested, closed-ended questions  2)patients as well as parents/siblings as respondents  3)prior knowledge on OMIs of 12.7% (21/165) of respondents  4)numbers and demographics of patients in the 2 different settings were not reported  5)risk of selective reporting, because no protocol was registered or published a priori |
| 10)Were subpopulations identified using objective criteria ? | NA | Subpopulations were identified, i.e., patients versus sibblings/parents or patients with prior knowledge or patients in different settings etc. However, prevalence data on implementation constructs of these subgroups were not reported. |

*Critical appraisal scores: ☺ Yes ☹ No ? Unclear NA Not Applicable

**Table 2. The Joanna Briggs Institute critical appraisal tool [35,59] of prevalence and incidence data for the study by Meeran et al. (2012)[8]* ****

| **Appraisal questions** | **Appraisal scores** | **Comments** |
| --- | --- | --- |
| 1)Was the sample representative of the target population ? | ☺ | The sample was not described in great detail, but seemed representative of the target population. |
| 2)Were study participants recruited in an appropriate way ? | ? | We scored this domain as ‘unclear’, because no information was provided on the selection procedures. For example why was a sample of exactly 2100 selected. Are these all orthodontists practicing in India etc.? |
| 3)Was the sample size adequate ? | ☺ | Sample size was adequate |
| 4)Were the study subjects and the setting described in detail ? | ? | The sample was not described in great detail. The sum of the non-users in the different settings (972) is different from the sum of the non-users (952) mentioned in the results |
| 5)Was the data analysis conducted with sufficient coverage of the identified sample ? | ☺ | The overall response rate was good: 1691/2100 = 0.80523 |
| 6)Were objective, standard criteria used for the measurement of the condition ? | ☹ | No reference was made to a reference standard for assessing implementation constructs. Survey instruments were not validated. |
| 7)Was the condition measured reliably ? | ☺ | Yes, only closed-ended questions |
| 8)Was there appropriate statistical analysis ? | ☺ | Yes, only prevalence data were presented with appropriate numerators and denominators |
| 9)Are all important confounding factors/subgroups/differences identified and accounted for ? | ☹ | Confounding factors/subgroups included:  1)non-defined, not-pilot-tested, closed-ended questions  2) The sum of the non-users in the different settings (972) is different from the sum of the non-users (952) mentioned in the results  3)Urban respondents were overrepresented in the total sample and had less non-users compared with the non-urban population**  4)risk of selective reporting, because no protocol was registered or published a priori |
| 10)Were subpopulations identified using objective criteria ? | NA | Subpopulations were identified, i.e., urban and non-urban settings. However, prevalence data on implementation constructs of these subgroups were not reported. |

* Critical appraisal scores: ☺ Yes ☹ No ? Unclear NA Not Applicable

****Users and non-users of OMIs in different settings**

| **Setting** | **Total** | **% of total** | **Users of OMIs** | **Non-users of OMIs** | **Non-users of OMIs %** |
| --- | --- | --- | --- | --- | --- |
| **Urban** | 988 | 57.7% (988/1711) | 537 | 451 | 45.6% (451/988) |
| **Non-Urban** | 723 | 42.3% (723/1711) | 202 | 521 | 72.1% (521/723) |
| **Total** | 1711 | 100% | 739 | 972 | 100% |

**Table 3. The Joanna Briggs Institute critical appraisal tool [35,59] of prevalence and incidence data for the study by Bock et al. (2015)[5]***

| **Appraisal questions** | **Appraisal scores** | **Comments** |
| --- | --- | --- |
| 1)Was the sample representative of the target population ? | ? | The 439 non-users of OMIs were adequately described, but included both 417 (95%) strict non-users of OMIs and 22 (5%) osseointegrated palatal implants users. Only strict non-users of OMIs would have been our preferred target population. |
| 2)Were study participants recruited in an appropriate way ? | ☺ | No limitations were identified with respect to the recruiting procedures |
| 3)Was the sample size adequate ? | ☺ | The sample sizes of the non-users of OMIs (439) and the 345 respondents were adequate. |
| 4)Were the study subjects and the setting described in detail ? | ? | The 439 non-users and their settings were adequately described, but the characteristics and settings of the 345 respondents on implementation constructs of these 439 non-users were not described. |
| 5)Was the data analysis conducted with sufficient coverage of the identified sample ? | ? | The response rate of the non-users of OMIs was 78.6% (345/439). The 439 non-users were adequately described. However, the rationale and the consequences of this dropout and the characteristics of the 345 respondents on implementation constructs were not described. |
| 6)Were objective, standard criteria used for the measurement of the condition ? | ☹ | No reference was made to a reference standard for assessing implementation constructs. Survey instruments were not validated. |
| 7)Was the condition measured reliably ? | ☺ | Yes, only closed-ended questions |
| 8)Was there appropriate statistical analysis ? | ☺ | Yes, only prevalence data were presented with appropriate numerators and denominators. These statistics were not completely clear in the published article and were confirmed through contacting the authors of the research study. This domain was subsequently upgraded from ‘Unclear’ to ‘Yes’. |
| 9)Are all important confounding factors/subgroups/differences identified and accounted for ? | ☹ | Confounding factors/subgroups included:  1)non-defined, not-pilot-tested, closed-ended questions  2) Both strict non-users of OMIs and users of osseointegrated palatal implants were included among the respondents of non-users of OMIs.  3)Various types of experience with OMIs among subgroups of non-users of OMIs.  4) The 439 non-users were adequately described, but the characteristics of the 345 respondents on implementation constructs of these 439 non-users were not described.  5)risk of selective reporting, because no protocol was registered or published a priori. |
| 10)Were subpopulations identified using objective criteria ? | NA | There were 2 subgroups of non users: Strict non-users and OPI users. They were identified using objective criteria. However, prevalence data on implementation constructs of these subgroups were not reported. |

* Critical appraisal scores: ☺ Yes ☹ No ? Unclear NA Not Applicable
