# Supplementary material for: Barriers and facilitators to the implementation of orthodontic mini implants in clinical practice: a systematic review
Source: Syst Rev. 2016 Sep 23;5:163. doi: 10.1186/s13643-016-0336-z (PMC5034676; doi:10.1186/s13643-016-0336-z)
Supplement: Additional file 5: — Questions for contacted authors and outcomes. (DOCX 28 kb) [file 13643_2016_336_MOESM5_ESM.docx]

**Additional file 5.**

**Questions for contacted authors and outcomes**

**Table 1. Outcomes of author’s willingness to reply to questions of systematic reviewers**

| **Article** | **Contacted author(s)** | **Number of contacting attempts and time to get a response** | **Reminder mails?** | **Co-authors contacted?** | **Willingness to reply ?** |
| --- | --- | --- | --- | --- | --- |
| Zawawi 2014[85] | Dr. Zawawi | 1 attempt  Response within 1 day | No | No | Yes |
| Meeran  2012[8] | Dr. Meeran and Dr. Venkatesh | Total of 3 attempts  2 attempts to contact Dr. Meeran, but he did not respond.  Dr Venkatesh responded within 1 day after being contacted | Yes | Yes | Yes |
| Bock 2015[5] | Dr. Bock | 1 attempt  Response within 1 day | No | No | Yes |

**Table 2. Outcomes of answering by contacted authors to research questions of systematic reviewers**

| **Article** | **Contacted author(s)** | **Number of contacting attempts to get a reply to research questions** | **Reminder mails?** | **Co-authors contacted?** | **Number of research questions answered** |
| --- | --- | --- | --- | --- | --- |
| Zawawi 2014[85] | Dr. Zawawi | 1 attempt  Response within 1 day | No | No | 9 of 9 |
| Meeran  2012[8] | Dr. Meeran and Dr. Venkatesh | Total of 4 attempts  2 attempts to contact Dr. Meeran and 2 attempts to contact Dr. Venkatesh, but they both did not respond to our research questions. | Yes | Yes | None |
| Bock 2015[5] | Dr. Bock | 1 attempt  Response within 1 day | No | No | 5 of 5 |

**Table 3. Questions for the study by Zawawi (2014)[85]**

| **Questions** |
| --- |
| **Question 1.**  Was a protocol of your research study developed prior to starting the study?  If you answer with a YES, could you please describe whether this protocol was registered or published and in what register or journal?  If you answer with a NO, it is not necessary to provide any additional information. |
| **Question 2.**  Were all patients that soughed orthodontic treatment (page 934 column 1) included in the survey, in other words were these patients consecutively treated, e.g. all patients seeking orthodontic treatment between specific dates?  If you answer with a YES, could you please describe how this procedure was conducted?  If you answer with a NO, it is not necessary to provide any additional information. |
| **Question 3.**  Was a power calculation conducted to determine the adequate sample size?  If you answer with a YES, could you please describe how this procedure was conducted and the outcome of this calculation?  If you answer with a NO, it is not necessary to provide any additional information. |
| **Question 4.**  Were the questionnaires completed prior to the interventional procedures with orthodontic miniscrews?  If you answer with a YES, could you please describe when these questionnaires were completed?  If you answer with a NO, it is not necessary to provide any additional information. |
| **Question 5.**  Did the patients that completed Questionnaire 1 (all 165 patients) knew beforehand (before completing the questionnaire) whether they needed miniscrews or not (page 934 column 2)? This refers to the section where you describe that 83 (29 men and 54 women) were going to have miniscrews as part of their treatment. The other 82 had orthodontic treatment without miniscrews (page 934 column 2).  If you answer with a YES, could you please explain?  If you answer with a NO, it is not necessary to provide any additional information. |
| **Question 6.**  You describe that certain patients had prior knowledge in the sense that they had heard about miniscrews before.  12.7 % had prior knowledge of the screws (on page 936 column 1) (Table 1). Did some of these patients had previously undergone orthodontic treatment with miniscrews or did they just heard about it?    If you answer with a YES, could you please explain?  If you answer with a NO, it is not necessary to provide any additional information. |
| **Question 7.**  Where was the private office located (also in Jeddah ?) to which you refer on page 934 column 1.  If you answer with a YES, could you please explain?  If you answer with a NO, it is not necessary to provide any additional information. |
| **Question 8.**  Could you provide some additional information on the different settings, i.e., differences between university and private practice patients ? For example we would like to know the number of patients from each setting ? If you have easy access to some additional data, we would also be very pleased to receive some information on the age, sex, and demographics of the patients of these different settings.  If you answer with a YES, could you please explain?  If you answer with a NO, it is not necessary to provide any additional information: |
| **Question 9.**  Was the response rate 100%, i.e. 165 of 165 answered the questionnaires ?    If you answer with a YES, could you please explain?  If you answer with a NO, it is not necessary to provide any additional information. |

**Table 4. Questions for the study by Bock et al. (2015)[5]**

| **Questions** |
| --- |
| **Question 1.**  Was a protocol of your research study developed prior to starting the study?    If you answer with a YES, could you please describe whether this protocol was registered or published and in what register or journal?  If you answer with a NO, it is not necessary to provide any additional information. |
| **Question 2.**  Was a power calculation conducted to determine the adequate sample size?    If you answer with a YES, could you please describe how this procedure was conducted and the outcome of this calculation?  If you answer with a NO, it is not necessary to provide any additional information. |
| **Question 3.**  Do the Osseo-integrated palatal implants refer to implants with diameters larger than 2.5 mm?    If you answer with a YES, could you please describe the diameter of these implants?  If you answer with a NO, it is not necessary to provide any additional information. |
|  |

| **Question 4.**  We know that there are 439 non-users of MSCs (417 strict non-users and 22 OPI-only users)(Table 3). Of these 439 participants there were 345 boxes checked on the rationale for non-use of miniscrews.  However, we like to know: **What is the response rate among MSC non-users ?** In other words we want to know how many of the 439 MSC non-users checked one or more boxes of the Reasons for non-use of MSCs/OPIs questionnaire ? Note we are only interested in the numbers for the MSC non-users group and NOT in the OPI non-user group !!!  **You would help us even more when you completed the following table, i.e. inserting the missing numbers that are now marked as ??**     \| **Boxes with reasons for non use** \| **Total number ?? of MSC non-users that checked boxes** \| \| --- \| --- \| \| (Almost) no suitable indications within treatment concept \| Number ?? of MSC non-users that checked this box /Total number ?? of MSC non-users that checked boxes \| \| Skeptical about there being an additional benefit \| Number ?? of MSC non-users that checked this box /Total number ?? of MSC non-users that checked boxes \| \| Skeptical about their success/failure rate \| Number ?? of MSC non-users that checked this box /Total number ?? of MSC non-users that checked boxes \| \| Insertion/surgery too complex or time-consuming \| Number ?? of MSC non-users that checked this box /Total number ?? of MSC non-users that checked boxes \| \| Concerned about their complication/injury rate \| Number ?? of MSC non-users that checked this box /Total number ?? of MSC non-users that checked boxes \| \| No appropriate surgeon nearby \| Number ?? of MSC non-users that checked this box /Total number ?? of MSC non-users that checked boxes \| \| Insertion surgery too expensive \| Number ?? of MSC non-users that checked this box /Total number ?? of MSC non-users that checked boxes \|     **Question 5.**    **Please note:** If for some reason you are unable to present the missing numbers in table above, could you then **please explain how you calculated the percentages for the Reasons for non-use of MSCs/OPIs, i.e. 51%, 33.3%, 56.2%, 32.5%, 24.3%, 32.8%, and 9.6% ?** |
| --- | --- | --- | --- | --- | --- | --- | --- | --- | --- | --- | --- | --- | --- | --- | --- | --- |

**Table 5. Questions for the study by Meeran et al. (2012)[8]**

| **Questions** |
| --- |
| **Question 1.**  Was a protocol of your research study developed prior to starting the study?  If you answer with a YES, could you please describe whether this protocol was registered or published and in what register or journal?  If you answer with a NO, it is not necessary to provide any additional information. |
| **Question 2.**  A survey questionnaire was sent to 2100 qualified orthodontists practicing in India (Page 46 column 2). Are these all the qualified orthodontists in India or was this a subgroup of orthodontists ? If this was a subgroup, could you describe this subgroup of 2100 qualified orthodontists and how it was selected? (Page 46 column 2) |
| **Question 3.**  Was a power calculation conducted to determine the adequate sample size?  If you answer with a YES, could you please describe how this procedure was conducted and the outcome of this calculation?  If you answer with a NO, it is not necessary to provide any additional information |
| **Question 4.**  We would like to get some clarification on the response rate. A survey questionnaire was sent to 2100 qualified orthodontists practicing in India (Page 46 column 2). A total of 988 respondents practiced in an urban setup while 723 had practice in a semi urban or rural locality (Page 47 column 1). This counts for a total of 1711 respondents. However, on the same page (and also in the abstract) you write that 952 never used miniscrews and 739 had used them. This counts for a total of 1691 respondents.  Could you explain  1)  which is the correct total number of respondents?  2)  and how this total number is divided between users and non-users?  3)  and how this number is divided between urban and semi urban or rural settings ? |
| **Question 5.**  The reasons given by orthodontists for not using miniscrews in their clinical practice were described on page 47 first column and are summarized in table 4. Are the percentages in this table based on the answers of both users and non-users of miniscrews (739 + 952 respondents) or only based on the answers of the non-users of miniscrews (952 respondents)? |
